# Putting hornets on the genomic map

**DOI:** 10.1038/s41598-023-31932-x

**Published:** 2023-04-21

**Authors:** Emeline Favreau, Alessandro Cini, Daisy Taylor, Francisco Câmara Ferreira, Michael A. Bentley, Federico Cappa, Rita Cervo, Eyal Privman, Jadesada Schneider, Denis Thiéry, Rahia Mashoodh, Christopher D. R. Wyatt, Robert L. Brown, Alexandrina Bodrug-Schepers, Nancy Stralis-Pavese, Juliane C. Dohm, Daniel Mead, Heinz Himmelbauer, Roderic Guigo, Seirian Sumner

**Affiliations:** 1 Centre for Biodiversity and Environmental Research, Department of Genetics, Evolution and Environment, University College London, Gower Street, London, WC1E 6BT UK; 2 Department of Biology, Università di Pisa, Via Volta 6, 56126 Pisa, Italy; 3 Centre for Genomic Regulation, Dr. Aiguader 88, 08003 Barcelona, Spain; 4 Department of Biology, University of Florence, Via Madonna del Piano 6, 50019 Sesto Fiorentino, Florence, Italy; 5 Department of Evolutionary and Environmental Biology, Institute of Evolution, University of Haifa, Abba Hushi 199, 3498838 Haifa, Israel; 6 INRAe, UMR 1065 Santé et Agroécologie du Vignoble, Bordeaux Sciences Agro, ISVV, Université de Bordeaux, 33883 Villenave d’Ornon, France; 7 Manaaki Whenua - Landcare Research, 54 Gerald Street, Lincoln, 7608 New Zealand; 8 Department of Biotechnology, Institute of Computational Biology, University of Natural Resources and Life Sciences, Vienna, Muthgasse 18, 1190 Vienna, Austria; 9 Tree of Life Programme, Wellcome Sanger Institute, Hinxton, CB10 1SA UK; 10 Universitat Pompeu Fabra, Barcelona, Spain

**Keywords:** Ecology, Molecular biology

## Abstract

Hornets are the largest of the social wasps, and are important regulators of insect populations in their native ranges. Hornets are also very successful as invasive species, with often devastating economic, ecological and societal effects. Understanding why these wasps are such successful invaders is critical to managing future introductions and minimising impact on native biodiversity. Critical to the management toolkit is a comprehensive genomic resource for these insects. Here we provide the annotated genomes for two hornets, *Vespa crabro* and *Vespa velutina.* We compare their genomes with those of other social Hymenoptera, including the northern giant hornet *Vespa mandarinia*. The three hornet genomes show evidence of selection pressure on genes associated with reproduction, which might facilitate the transition into invasive ranges. *Vespa crabro* has experienced positive selection on the highest number of genes, including those putatively associated with molecular binding and olfactory systems. Caste-specific brain transcriptomic analysis also revealed 133 differentially expressed genes, some of which are associated with olfactory functions. This report provides a spring-board for advancing our understanding of the evolution and ecology of hornets, and opens up opportunities for using molecular methods in the future management of both native and invasive populations of these over-looked insects.

## Introduction

Insects are the most speciose and abundant organisms on the planet. This is an exciting time for insect research as we are enjoying an explosion in the sequencing of insect genomes^1,2^. At the time of writing, 3058 insect genomes have been published on International Nucleotide Sequence Database Collaboration (INSDC); although impressive, this represents only 0.1% of described insect species. Having a genome sequenced for a species opens up a wealth of research opportunities with benefits to evolutionary biologists, ecologists and conservation scientists^3^. Genomic resources provide clues to understand how and why species distributions are affected by anthropogenic actions, and to develop effective ways to track and manage populations. This is especially important for ecologically and economically important species, which provide critical ecosystem services on which planetary health depends (e.g., crop pest control, biodiversity and agriculture pollination); but it is also important for managing species which become problematic outside of their native ranges, as invasive species. The social insects (termites, ants, some bees and wasps) account for 75% of the insect biomass^4^; ant biomass alone equals 20% of human biomass^5^. It is not surprising, therefore, that this group of insects has amongst the greatest ecological and economic impact on natural and farmed ecosystems^6^. This, together with their fascinating social lives and relatively small genomes, has made the social insects popular choices for genome sequencing projects. Indeed, there are more species of the social Hymenoptera (ants, bees, wasps) with published genome sequences (National Center for Biotechnology Information (NCBI) RefSeq reference genomes or 158 species at the time of writing) than there are for the more highly speciose insect orders such as Coleoptera (beetles) (NCBI RefSeq reference genomes for 110 species at the time of writing). Amongst the insects, the number of hymenopteran genomes sequenced is second only to the Diptera^1^. Despite this focus, there remains a critical taxonomic bias^7^, with 89% of the sequenced hymenopteran genomes belonging to ants and bees, leaving a mere 11% Vespidae wasp species genomes on INSDC (including nine social wasp and hornet species; Supplementary Table ST1). The paucity of genomic resources for wasps is perplexing as they provide critical ecological services as regulators of arthropod populations, pollinators, seed dispersers and as a popular source of human nutrition in some parts of the world (e.g. Japan; China; South America)^8^. Moreover, the wasps include several of the world’s worst invasive species^9–11^. There is an urgent need to widen the genomic resources available for these important facets of our planet’s biodiversity.

Insect genome sequencing has been increasingly popular thanks to significant technological advances such as long-read sequencing^12^ and three-dimensional chromosomal mapping methods^13^. Substantial funding initiatives contribute to this genome production line, such as the Darwin Tree of Life project, which aims to generate high-quality genomes from all eukaryote species in Britain and Ireland^14,15^, the i5K Consortium which aims to sequence 5000 high-priority arthropod species, including the top 100 agricultural pests in the US^16^, and the Global Ant Genomic Alliance which is sequencing high-quality genomes for 200 ant species^17^. Social insect genomes improve our understanding of the evolution of social organisation^7,18^, e.g. by identifying loci involved in communication, such as gene family expansions of ant and bee odorant receptors and termite lipocalins^19–21^, as well as improving our understanding of their ecology and behaviour, e.g. on how bees adapt to living at high altitudes^22^. Furthermore, genomic data can provide insights into biodiversity trends associated with climate change and invasive species^23–25^.

There are around 1,200 species of social wasps, including relatively well-known Vespidae yellowjackets and hornets^26^, and lesser-known species of Stenogastrinae^27^ and Polistinae^28^. They display a huge variety of ecological and life-history characteristics; e.g. the colony size varies enormously, from species with less than 10 individuals in the society (e.g. as found in the Stenogastrine hover wasps) to those with tens of thousands of workers (e.g. *Vespula* species); some species have many reproductive queens (e.g. epiponine wasps, like *Metapolybia)* whilst others are monogynous (e.g. *Vespa crabro)*; reproductive hierarchies can be regulated by conventions such as age or size^29^, aggression^30^ or pheromones^31^.

**Figure 1:**
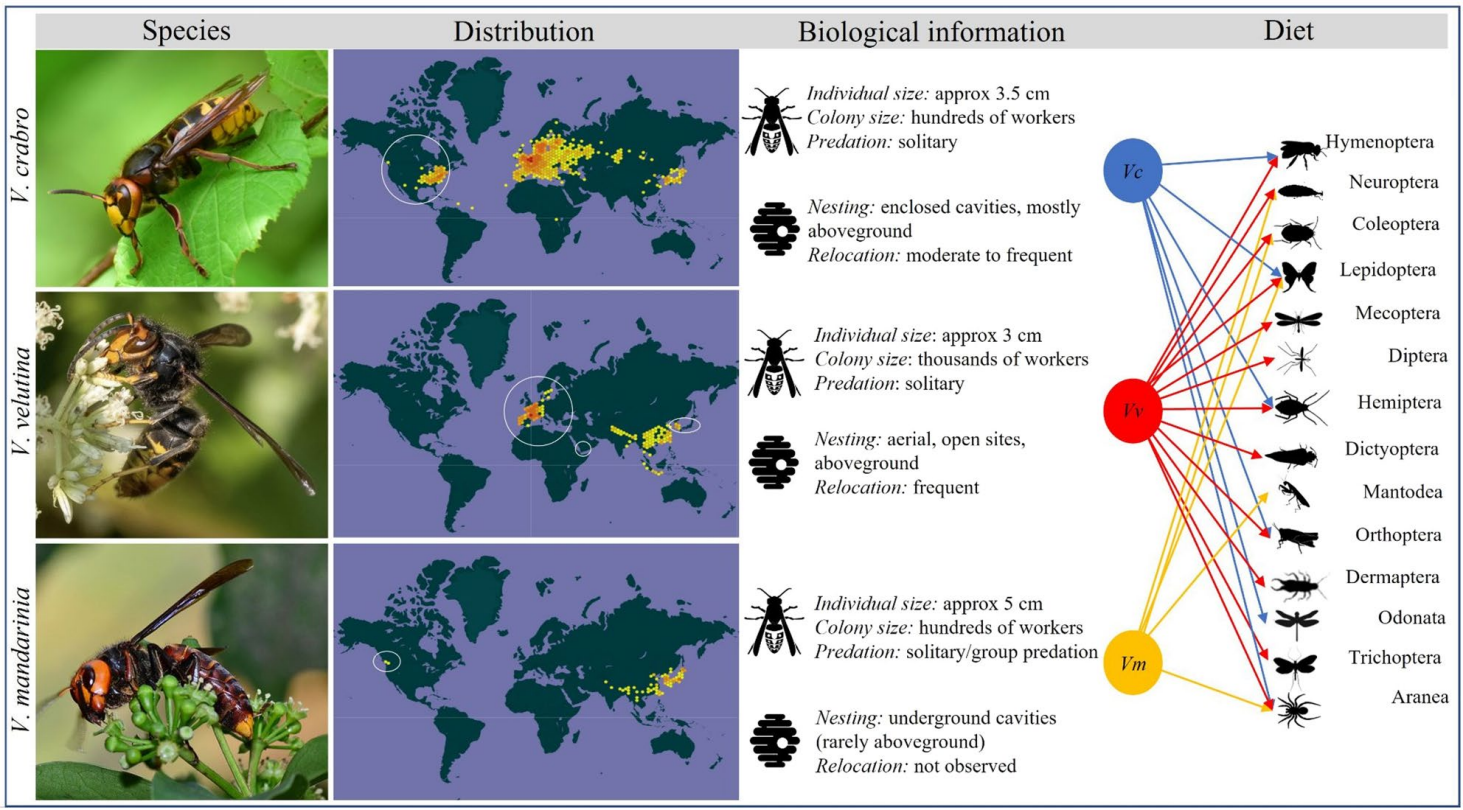
Biology of *Vespa* hornets. Comparing the key life-history traits of the three *Vespa* species analyzed in this study. Species column: Female adult morphology for *Vespa crabro*, *Vespa velutina* and *Vespa mandarinia* (photos taken from www.inaturalist.org, respectively from the following users: rainyang, Mиxaил Maлышeв, Kinmatsu Lin, all photos have CCBY-NC license). Distribution column: Known geographical distribution of species (from https://www.gbif.org/)^40–42^; the redder patches indicate higher occurrence records; their invasive distributions are circled. Biological information column: Descriptions of individual and nest traits and behaviours^28,37,39,43^. Diet column: All three *Vespa* species (left) prey on a diverse set of arthropod orders (right)^8,44,45^.

The first aculeate wasp genome was published in 2015 (*Polistes canadensis*^32^), closely followed by the European paper wasp *Polistes dominula*^33^. Currently there are genome sequences published for seven polistine wasp species and nine vespine wasps (including those presented in this paper; see Table 1). However, evolutionary analyses of these vespine genomes are lacking, and particularly so for the hornets, the *Vespa* genus. Such analyses are important for several reasons (Fig. 1). *Vespa* exhibit some of the most complex societies of the aculeate wasps, with colonies headed by one (or a few) queens who are morphologically distinct from their workers^34^. They constitute an important part of our planet’s natural capital as top predators and regulators of insect populations, pollinators and seed dispersers^8^. Several species of *Vespa* have been inadvertently introduced outside of the native range where their colony life span can be longer and they have become problematic as invasive species^35^; for instance the European hornet *Vespa crabro* has become established in the USA^34^. Some invasions threaten local fauna; e.g. the recent invasion of *Vespa velutina* from South East Asia threatens beekeeping in Europe^36–38^*; Vespa mandarinia* from South East Asia was introduced in several parts of North America where it poses a threat to local ecosystems and human health^39^. Given their ecological and economic importance and their history of invasion success, it is surprising that *Vespa* have not received more attention as subjects of genomic studies. It is therefore time to put *Vespa* genomes on the map and explore their potential.

**Table 1:** Comparison of hornet (*Vespa)* genome assemblies statistics with honeybee, fire ant and other social wasp genomes.

Species	Family	Subfamily	Size (bp)	Scaffolds	N50	L50	GC%	# N’s per 100 kbp
Vespa crabro*	Vespidae	Vespinae	211,313,510	30,304	34,273	1021	32.19	15,781
Vespa velutina*	Vespidae	Vespinae	193,976,845	42	9,190,824	8	32.86	50
*Vespa mandarinia*^46^	Vespidae	Vespinae	247,731,252	268	2,778,186	26	30.55	0
*Vespula germanica *^76^	Vespidae	Vespinae	205,789,424	37	9,441,317	8	35.13	3
*Vespula pensylvanica*^76^	Vespidae	Vespinae	179,370,016	222	8,532,720	8	34.39	248
*Vespula vulgaris*^76^	Vespidae	Vespinae	188,204,803	28	8,749,684	8	34.62	3
*Polistes canadensis*^32^	Vespidae	Polistinae	211,202,212	3836	521,566	103	32.15	6689
*Polistes dominula*^33^	Vespidae	Polistinae	208,026,220	1483	1,625,592	37	30.77	3588
*Polistes dorsalis*^77^	Vespidae	Polistinae	209,288,276	5129	5,372,633	13	32.54	2280
*Polistes fuscatus*^77^	Vespidae	Polistinae	219,116,742	187	9,116,088	8	32.74	2025
*Polistes metricus*^77^	Vespidae	Polistinae	219,838,961	216	1,605,847	14	32.35	493
*Polistes exclamans*^75^	Vespidae	Polistinae	206,639,3415	1793	4,110,000	17	32.06	NA
*Mischocyttarus mexicanus*^75^	Vespidae	Polistinae	212,903,210	3793	1,100,000	41	32.4	NA
*Apis mellifera*	Apidae	Apinae	225,250,884	177	13,619,445	7	32.53	583
*Solenopsis invicta*	Formicidae	Myrmicinae	396,009,169	45,178	14,674	131	36.18	10,642

The three *Vespa* assemblies (including our two assemblies with the star) with recent wasp genomes and one representative from each ant and bee group. All data in Supplementary Table ST4.

Here we compare the genomes of three ecologically, economically and societally important hornets—the European hornet *Vespa crabro,* the yellow-legged Asian hornet *Vespa velutina*, and the northern giant hornet *Vespa mandarinia*^46^*.* Two of these genomes (*V. crabro* and *V. velutina*) were sequenced and annotated for the purposes of this study. We compare the genome compositions of the three *Vespa* species with each other and contrast them with other social insect genomes (Aim 1). We are specifically interested in comparing lineages of insects that have evolved superorganismality independently^47^, to explore losses and gains of genes related to social organisation, such as chemoreceptors^48,49^. We identified genes undergoing rapid evolution and family expansions (Aim 2) and identified specific differences among the three *Vespa* species, such as duplication events among genes involved in communication. These provide insights into the ecological differences among the three study species, for instance by describing species-specific olfactory systems. We identified signs of positive selection in *Vespa* relative to other social insects and determined whether they were enriched for any specific functionalities. We found little evidence that genes under selection may be involved in caste determination. Finally, we examined differential gene expression among castes in one species, *V. crabro* (Aim 3), presenting the first transcriptomic analyses of castes in a superorganismal wasp.

## Materials and methods

### Sample collection

For Aim 1, four colonies of *V. crabro* were collected in September 2017 from four different locations in their native range of southern England (see Supplementary Table ST2). Individual workers, gynes (non-mated queens), males and queens were flash frozen on dry ice or collected straight into RNAlater and stored at – 80 °C thereafter. Samples from two colonies of *V. velutina* were collected in 2017 from Ventimiglia, Italy which is part of the invasive range^50^. Workers and gynes were collected alive and stored immediately in ethanol and − 20 °C thereafter.

### DNA and RNA library preparation and sequencing

DNA was extracted from whole bodies of two males of *V. crabro* using Qiagen DNeasy blood and tissue kit (Catalogue number: 69504; Hilden, Germany) following the manufacturers’ protocol (details of this and all following protocols in Supplementary Information). One sample contributed to the two runs from a pair-end TruSeq Nano LT library (Cat #: FC-121-4001, Illumina, San Diego, CA, USA; INSDC accession numbers: SRR11213735 and SRR11213736) and the other to the Nextera mate-pair library (Cat #: FC-132-1001, Illumina; SRR11213734). Sequencing was performed at the Vienna BioCenter Core Facilities on an Illumina HiSeq 2500 sequencer.

DNA was extracted from one male of *V. velutina* using Qiagen MagAttract HMW DNA extraction kit (Cat # 67563, Illumina). It was sequenced by the Wellcome Sanger Institute as part of their 25 genomes initiative (https://www.sanger.ac.uk/collaboration/25-genomes-for-25-years/, INSDC accession ERS3567203), producing a combination of Pacific Biosciences CLR (PacBio Express Library Kit Cat # 102-088-900; on needle-sheared DNA), 10 × Genomics Chromium and Hi-C data.

RNA was extracted from a range of tissues (from workers, gynes, queens and larvae) for gene annotation purposes. For Aim 3, we also sequenced brain RNA from two different castes—gynes and workers—of *V. crabro*. Using gynes rather than mature queens avoids the effects of matedness, age, overwintering, and reproductive maturity (developed eggs) on gene expression. We focused on measuring variation in gene expression related to behaviour between castes^47^; thus, brains were dissected from individuals derived from three to four different nests (see Supplementary Table ST2), and total RNA was extracted from individuals following published methods^51^ (RNeasy Mini Kit Cat # 74104, Qiagen). RNA extractions were checked for quality using TapeStation. Three to four brains were then pooled for each caste, ensuring that no single pool had more than one wasp from the same nest; this generated a total of four worker pooled samples and five gyne pooled samples for sequencing, representing respectively four and five biological replicates. We sequenced these nine pooled samples (150 bp paired-end, Illumina NovaSeq platform, by Novogene) and obtained at least 22 million reads per sample (Supplementary Table ST2).

### Genome assembly and annotation

We assembled and annotated two new *Vespa* genomes. The genome of *V. crabro* was assembled with SOAPdenovo_v2.04^52,53^ into contigs from one haploid male. The resulting assembly was screened for contaminants during INSDC submission (INSDC accession: JAITYU000000000). The genome of *V. velutina* (INSDC accession PRJEB46979) was assembled with the following process, palindromic read correction with pbclip, initial PacBio assembly generation with Falcon-unzip^54^, retained haplotig separation with purge_dups, Hi-C based scaffolding with SALSA2, Arrow polishing (from Pacific Biosciences, https://github.com/PacificBiosciences/GenomicConsensus), and short-read polishing using FreeBayes-called variants from 10× Genomics Chromium reads aligned with LongRanger. Chromosome-scale scaffolds confirmed by the Hi-C data have been named in order of size.

In order to compare genome compositions amongst *Vespa* and with other social insects (Aim 2), we annotated these two new genomes for structural and functional loci. We used RNAseq data combined with “ab initio” and comparative computational methods, including mapping wasp UniProt-derived proteins, to predict genes in the repeatmasked genomes of *V. crabro* and *V. velutina* (Supplementary Information; Supplementary Table ST3). We obtained genome and annotation files from all other species from INSDC (Supplementary Table ST4). We estimated the assemblies’ statistics using BUSCO v5.1.2^55^ and QUAST v5.0.2^56^. Additionally, we compared synteny between genomes (Supplementary Table ST5A) with D-genies using Minimap v.2^57,58^. Finally, to annotate Transposable Elements (TE), we used RepeatModeler v2.0.4^59^ to generate a model of TEs for the *V. velutina* and *V. crabro* genomes separately. Consensus sequences were clustered and filtered using cd-hit v4.6.8^60^ and BBmap v39.01^61^ as recommended^62^. We then used RepeatMasker v4.1.0^63^ to annotate TEs based on the model for that genome (Supplementary Table ST4.

### Orthogroup exploration for positive selection and duplication events

**Figure 2:**
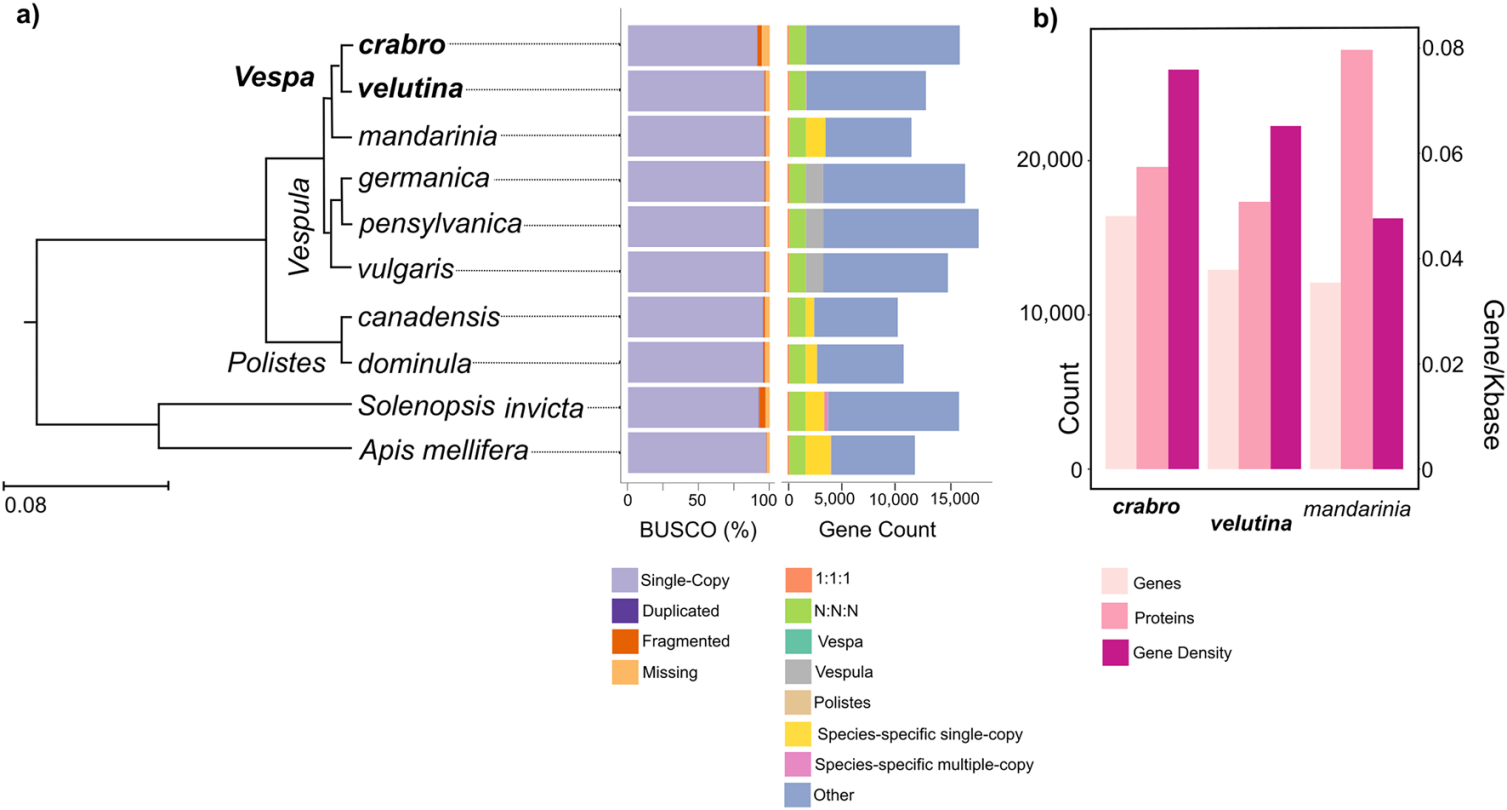
*Vespa* genomes statistics in Hymenoptera context. (**a**) Phylogenetic context and protein-coding gene content of the two new genomes (in bold) with *Apis* bee, *Solenopsis* ant, *Polistes* paper wasps, *Vespula* yellowjacket wasps. Branch lengths (unit: number of substitutions per site) are from species tree inference algorithm STAG (OrthoFinder). Left Bar Plot: Most Hymenoptera BUSCO genes are found as single copies (mauve) although a small number were duplicated (purple), fragmented genes (dark orange) or missing (light orange). *Vespa crabro* had a higher proportion of fragmented BUSCOs (Complete: 91.3% [Single-copy: 91.1%, Duplicated: 0.2%], Fragmented: 3.2%, Missing: 5.5%, n: 5991) than *Vespa velutina* (C: 96.3%[S: 96.1%, D: 0.2%], F: 0.9%, M: 2.8%, n: 5991) and *Vespa mandarinia* (C: 96.4%[S: 96.1%, D: 0.3%], F: 0.9%, M: 2.7%, n: 5991). Right Bar Plot: Total gene counts in each species in relation to OrthoFinder results. Out of 17,061 orthogroups, 163 are single-copy across the ten species (orange) and 1595 are found in multiple copies (green). Most of the protein-coding genes of each species are orthologous to one extend (Other, blue). (**b**) *Vespa* genomes composition: number of protein-coding genes, number of proteins, gene density (number of genes per 1000 bp), based on *V. crabro and V. velutina* EVM-consensus annotations, and based on *V. mandarinia* RefSeq annotation. All data in Supplementary Table ST4.

In order to explore patterns of gene evolution across the species (Aim 2), we ran OrthoFinder 2.5.4^64^ on the longest isoform of each protein, first on eight Hymenoptera species (*Vespa crabro, Vespa velutina, Vespa mandarinia, Vespula germanica, Vespula pensylvanica, Vespula vulgaris, Apis mellifera, Solenopsis invicta*; Fig. 2a), then on seven Hymenoptera species (without the ant, *S. invicta*). We based our duplication events exploration on the resulting listed multiple-copy orthologues for each species and each internal node.

We kept 2685 resulting nearly-1:1 orthogroups (i.e. between one and three copies of the gene in any given species, and any given gene had to be present in a minimum of six species) for the six wasps and the bee. This is a more relaxed filtering method than strict single-copy orthologs, allowing more data points in our exploration^49,65^. We then aligned all species’ sequences using PRANK v.151120 (gffread -x, PRANK options -f = paml -F -codon^66^). We investigated evidence of positive selection being experienced in the past on a specific branch (branch-test; M0 (Model = 0, Nsites = 0); M2 (Model = 2, Nsites = 0)) with PAML v.l4.9j^67^ using codeml with the phylogenetic tree from OrthoFinder of six vespid species (three Vespa and three Vespula species). To gain more insight on specific loci, we then ran branch-site models, focusing on potential loci on specific branches (“foreground”) against other branches (“background”) using codeml’s dN/dS branch-site models of positive selection^68^. Briefly, we compared the ratio of non-synonymous mutations to synonymous mutations (ω) in nearly 1:1 single-copy orthogroups of six those vespid species and tested two models: neutral evolution in foreground branch (Model = 2, Nsites = 2; ω fixed at 1, null hypothesis) and positive selection in foreground branch (Model = 2, Nsites = 2; ω > 1, alternative hypothesis; Supplementary Table ST6). We ran likelihood ratio tests^67^ and further tested for association with significance of log-likelihood test with chi square tests. Significant loci having experienced positive selection are those orthogroups with adjusted *P* value (Benjamini-Hochberg) below 0.05. We then assigned a description to each orthogroup using BLASTp v.2.10.0^69^ with *A. mellifera* as query to the *nr* database. Finally, we obtained GO terms from our gene subsets using TopGO v.2.38.1^70^.

### Differential gene expression analysis

For Aim 3, we employed Nextflow pipeline nf-core/rnaseq v.19.10.0.5170^71^ to assess the quality of RNA reads with FastQC v0.11.8, to trim adapters with ‘TrimGalore!’ v0.6.4. We mapped the nine pooled samples (four gynes, five workers) against the genome with STAR vSTAR_2.6.1d^72^ and to obtain read count with featureCounts v1.6.4^73^. We assessed the quality of our data with a Principal Components Analysis of normalised read count, which showed samples to cluster by caste (Supplementary Figure SF7). We then conducted a differential gene expression analysis between worker and gyne read count using DESeq2 v.1.26.0^74^ (log normalization; alpha cutoff = 0.05; Bonferoni adjustment). Similar to the positive selection analysis, we used BLASTp and TopGO to explore results.

## Results and Discussion

### Aim 1: *Vespa* genomes statistics

We sequenced and assembled the draft genomes of *V. crabro* and *V. velutina*, using short-read and long-read data, respectively. We compare these assemblies to a publicly available assembly for *V. mandarinia* (Fig. 1), together with genomes from representatives of other aculeate wasps, ants and bees (Fig. 2a, Table 1). The *Vespa* genomes are similar in size (*V. crabro*: 211,313,510 bp, *V. velutina*: 193,994,974 bp, *V. mandarinia*: 247,710,421 bp; Table 1 and Supplementary Table ST4), and are within the expected range for Hymenoptera^7^. The degree to which the genome assemblies are fragmented reflects the sequencing technology used for each species. Those generated using short-read technology are more fragmented than those generated using long-read technology, and thus might influence our downstream report. The N50 for the short-read sequenced *V. crabro* was 34,273 bp, whilst that for the long-read sequences are significantly better (N50 for *V. velutina* was 9,190,824 bp; N50 for *V. mandarinia* was 2,778,186 bp; Table 1). All three *Vespa* species have low GC content (Table 1), as is characteristic of Hymenoptera^7^. *V. crabro* has 16,409 protein-coding genes and 19,597 annotated proteins; *V. velutina* has 12,928 protein-coding genes and 17,334 annotated proteins; the NCBI RefSeq annotation of *V. mandarinia* has 12,089 protein-coding genes and 27,185 annotated proteins (Fig. 2b); all of which are within known range for Hymenoptera (Supplementary Table ST4). The three *Vespa* assemblies have a high level of expected single-copy orthologous genes, as measured by the Hymenoptera BUSCO score (*V. crabro*: 91.1%, *V. velutina*: 96.1%, *V. mandarinia*: 96.1%; Fig. 2a and Supplementary Table ST4). These high BUSCO scores and the gene counts variation seen in the full-gene set barplot (Fig. 2a, Supplementary Table ST7) hint at lineage-specific differences. Indeed, there are only 14 genes common to the three *Vespa* species (in blue-green) whereas we counted 1,681 genes specific to the *Vespula* species (in grey). The genome of *Vespa crabro* contains a smaller proportion of TE (22%), compared to *Vespa velutina* (26%, Supplementary Table ST4; Supplementary Figure SF10). This range is larger than the 10% calculated in the Polistine lineage^75^. Both species’ TE landscape (Supplementary Figure SF8-SF9) fits within the expected range for insects. Interestingly, while all families appear to be larger in *V. velutina*, a larger proportion of Penelope elements are found in *V. crabro* (5.9%) compared to *V. velutina* (0.2%, which is within the range of recently published annotations of *Polistes exclamans* (0.16%) and *Mischocyttarus mexicanus* (0.02%)^75^). An extensive survey of Hymenoptera TEs will provide more insights in these variations.

In the pairwise assembly mapping between the three *Vespa* genomes, we find the highest contig similarity between *V. mandarinia* and *V. velutina* long-read genomes (88% of contigs are between 50 and 75% similar, Supplementary Table ST5A), while short-read *V. crabro* genome typically scores lower synteny score due to its fragmentation^34,57^. Interestingly, there is a large part of the *V. crabro* assembly that is not similar to the other two species’ assembly: over 5% of the *V. crabro* genome does not map to the *V. mandarinia* genome nor the *V. velutina* genome (*i.e.* no match in contig similarity; Supplementary Figures SF4–SF6), but these regions are small (less than 2000 bp) and not in coding regions (*i.e.* not in the annotation file). Their sequences are not similar to those of the closest model organism *Apis mellifera* (Supplementary Table ST5B)*;* they are most probably representing some repetitive elements. We additionally find some evidence of a one-off diagonal inversion in *V. mandarinia* (length: 1,152,777 bp; on original strand: locus NW_023395844.1, start 3,701,405, end 5,336,819) when mapped to *V. velutina* (on original strand: locus SUPER_22, start 2,372,742, end 4,003,498; Supplementary Figure SF6). This region has 96 annotated *V. mandarinia* genes and seems to be only inverted between *V. mandarinia* and *V. velutina*, hinting towards a *V. mandarinia*-specific rearrangement. Further improvements in assembly contiguity for *V. crabro* as well as further *V. mandarinia* population-level resequencing are needed to quantify and qualify both the unique region covering 5% of *V. crabro* genome and this potential inversion in *V. mandarinia*.

### Aim 2: evidence of gene duplication and site-specific positive selection in *Vespa*

**Figure 3:**
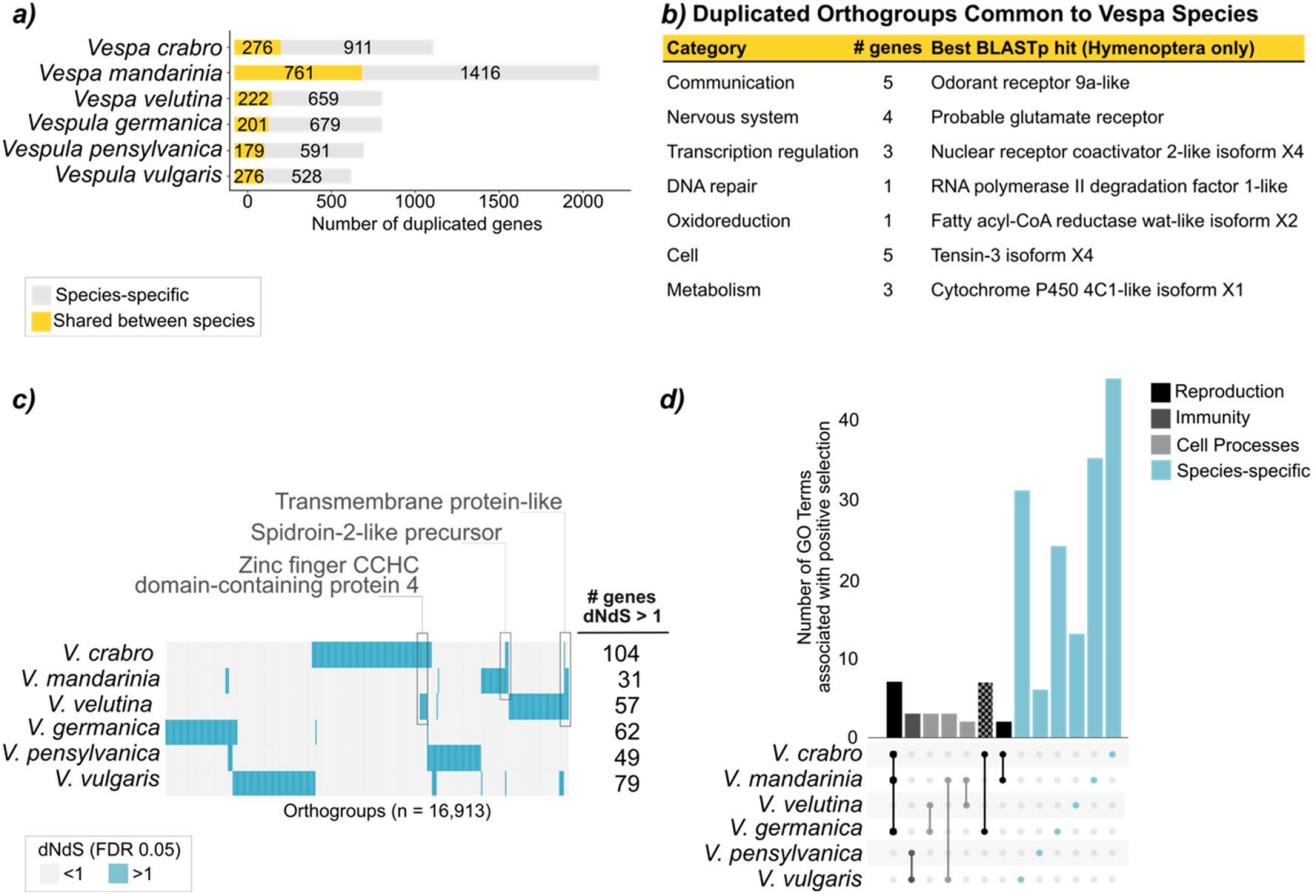
Comparative analyses of gene and protein evolution in vespine wasps. (**a**) Number of orthogroups with two or more copies, colour-coded by ancestral state (yellow: ancestral; grey: species-specific). *Vespa mandarinia* has the highest number of duplicated genes. All wasps have a high proportion of species-specific duplications. (**b**) Illustrative example of orthogroups that are duplicated in all three *Vespa* species, with associated Hymenoptera gene description after BLAST nr. (**c**) Orthogroups clustered by Euclidean distances of dN/dS categories (orthogroups in columns, blue represents positive selection in branch-site models) and by rows (species). There is very little overlap between species; overlapping areas with genes of interest are highlighted. Number of orthogroups that have experienced positive selection (dN/dS > 1, FDR 0.05) in *Vespa* and *Vespula* branches. *Vespa crabro* has the highest number of genes with a dN/dS ratio higher than 1 (n = 104). (**d**) Overlap of GO terms of orthogroups having experienced positive selection. Most of the species have a unique large set of GO terms, as seen in the tall, coloured bars on the far right-hand side of the graph (Fisher classic, unadjusted *P* value, see ST26–ST31.

Gene family expansions are thought to be associated with novel functions^78^ and invertebrate invasion^79^. Target gene families include those involved in communication, odorant binding^49^ and caste differentiation^80^. We thus compared the number of multiple copies of orthogroups between the three *Vespa* genomes. We focus on 16,913 orthogroups present in at least one species among ten chosen Hymenoptera representatives. Between lineages, we find more gene duplication events along the *Vespa* branch (25%; 5632 out of 22,976 events associated with 16,913 orthogroups) than along the *Vespula* branch (4%; 990 events; Supplementary Figure SF3). *V. crabro* has 1,187 duplicated orthogroups (7% of 16,913 orthogroups), including the highest proportion of species-specific gene family expansions: 77% of duplicated genes have two or more copies unique to this species (911 out of 1187). *V. mandarinia* has the highest number of duplicated orthogroups (2,177; 13% of 16,913 orthogroups), including 65% species-specific gene family expansions. *V. velutina* has 881 duplicated orthogroups (5% of 16,913 orthogroups), including 75% species-specific gene family expansions (Fig. 3a, Supplementary Table ST7). There are 28 genes that are duplicated in all three *Vespa*, some of which could be associated with the odorant or nervous systems (see notable examples of best BLASTp hits in Fig. 3b). *V. crabro*-specific gene duplications include sequences similar to notable Hymenoptera proteins. Those of particular interest include ten proteins from zinc finger family, a gene family known to also be duplicated in incipiently social *Ceratina* bees^80^; odorant receptors which are commonly found in lineage-specific expansions in Hymenoptera evolution^49^ and predicted to be duplicated in 80 invasive insect species^81^, and transposable element derived proteins which are involved in DNA-binding transcription factor activities and are thought to be associated with regulation of phenotypes via genome architecture variation^82^ (Supplementary Table ST8).

Selection pressures associated with social phenotypic plasticity impact the molecular evolution of proteins^83^. We thus explored the rate of protein evolution (dN/dS) for each single-copy orthogroup in the six *Vespa* and *Vespula* species (branch models: Supplementary Tables ST8–ST16; branch-site models: Supplementary Tables ST17-ST24). In branch-site models, *V. crabro* has 104 genes that showed evidence of having experienced positive selection (FDR 0.05, Fig. 3c, Supplementary Table ST17). From the best BLAST hits, notable loci include four zinc finger proteins (*e.g.* XP_006560154.1 zinc finger CCHC domain-containing protein 4)—a family known for its binding sites being more variable in social bees compared to solitary bees^84^ and known for being included in an ant social supergene^85^; an intraflagellar transport protein (XP_006560607.2)—known to be associated with insect olfactory sensilia used for communication^86^. In *V. velutina*, 57 genes showed evidence of positive selection (Supplementary Table ST21). Notable loci included: ELMO domain-containing protein C (XP_395400.7), found to be upregulated in a incipiently social bee when experimentally forced to increased brood provisioning^87^; and senecionine N-oxygenase (XP_006571261.2), which is involved in detoxification and found upregulated in pesticide-sprayed beetle^88^. In *Vespa mandarinia*, 31 genes showed evidence of positive selection (Supplementary Table ST19) including deformed epidermal autoregulatory factor 1 (XP_395757.3), a transcription factor associated with honey bee egg laying behaviour^89^. We found only one locus (OG0002464) that had experienced positive selection across all three *Vespa* species: the sequence matches in BLAST *Apis cerana*’s transmembrane protein-like (Fig. 3c). One locus—Spidroin-2-like precursor protein (NP_001314894.1)—was under selection in both *V. crabro* and *V. mandarinia;* this gene could be playing a role during pupa development^90^. Zinc finger CCHC domain-containing protein 4 (XP_006560154.1) was also under selection in both *V. crabro* and *V. velutina*.

When we compared these results with *Vespula* species, we found similar range of genes with evidence of positive selection (Supplementary Tables ST18, ST20, ST22). However, there was no overlap of genes with dN/dS > 1 between the six vespine wasp species nor distinct vespine chromosomal hotspot for positive selection (Fig. 3c in blue).

We next tested for GO term enrichment among the genes under positive selection in each *Vespa* branch-site model (Supplementary Tables ST25–ST30, Fig. 3d). There was no significant enrichment at the most stringent level (FDR 0.05). However, examination of terms with the lowest raw *P* values (*P*_*raw*_ < 0.05) revealed functions associated with reproduction and morphological innovations: oogenesis (GO:0048477, hallmark of eusocial species^91^) for *V. crabro*; hippo signalling (GO:0035329, involved in termite soldier morphological differentiation^92^) for *V. mandarinia*; signalling pathway (GO:0007224, part of the hedgehog signalling pathway associated with beetle horn morphological innovation^93^) for *V. velutina*. We additionally found species overlaps in GO terms, namely: cell signalling and reproduction in *Vespa* species; immunity in *Vespula* species.

In summary, the *Vespa* lineage may have experienced positive selection on genes related to communication, reproduction, production/regulation of alternative phenotypes. Our comparative analyses on orthologous genes consistently highlighted *V. crabro*, with the highest number of genes under positive selection and associated GO Terms, and with the highest proportion of species-specific duplicated genes in the *Vespa* lineage. Thus, we explored gene expression between phenotypes in this species, for our last Aim.

### Aim 3: caste-associated differential gene expression in *Vespa crabro*

**Figure 4:**
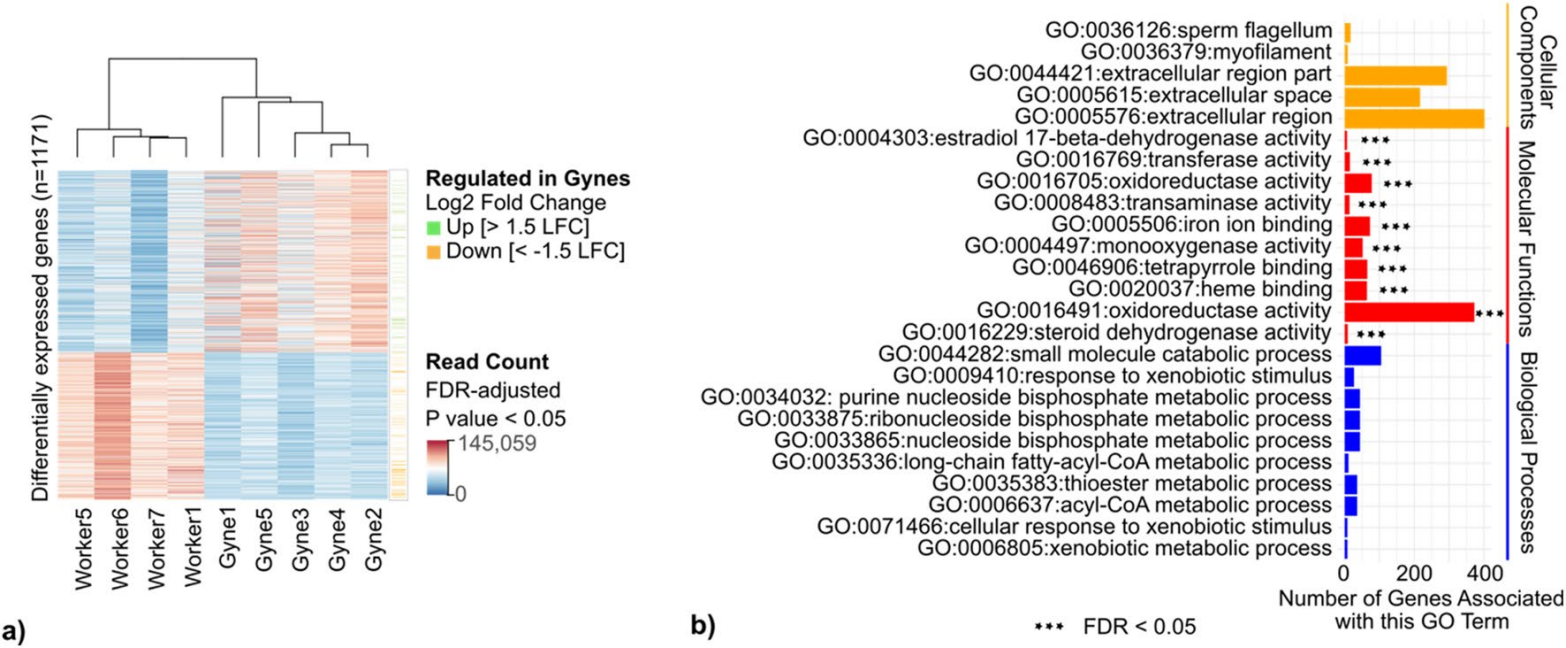
Caste-specific differentially expressed genes in *Vespa crabro* brain transcriptomes. (**a**) Differential expression analysis based on negative binomial distribution of read counts of 1171 genes from 4 workers and 5 non-mated queens (gynes) of *Vespa crabro*. Read counts from DESeq2 results are filtered by FDR adjusted *P* value < 0.05 and cluster by castes. Genes are colour-coded in the right-hand side column by stricter filtering (absolute log fold change above 1.5), resulting in 63 genes upregulated in gynes (green) and 70 downregulated (orange). (**b**) Top Gene Ontology Terms enriched in *Vespa crabro*’s differentially expressed genes (n = 133 DEG, FDR < 0.05, absolute log fold change = 1.5). 12 Molecular Function GO terms (red) are significantly enriched in the DEG.

Analyses of the molecular basis of caste differentiation in the brains of social insects has provided important insights into how division of labour in group living societies evolves^94,95^. However, to date these analyses in wasps have been limited to species from simple societies. Here we provide the first analysis of brain transcriptomes among castes of a superorganismal wasp. We extracted RNA from 40 individual brains of *V. crabro.* These were sequenced as five pooled samples of gynes (i.e. each sample contains brains from four individuals) and four pooled samples of workers (i.e. each sample contains brains from three individuals). We detected 1171 differentially expressed genes between workers and gynes (FDR-adjusted *P* value < 0.05; no log_2_ fold change filter; 648 upregulated, 523 downregulated, Fig. 4a). After filtering for log_2_ fold change 1.5, 133 genes remained: 63 were upregulated and 70 were downregulated genes in gynes relative to workers (Fig. 4b, Supplementary Table ST31). These included sequences with BLAST match to *Apis mellifera’*s zinc finger CCCH domain-containing protein 13—also found in *V. mandarinia*’s queen transcriptome^96^—, and odorant receptor proteins, recurrent in all hymenopteran olfactory repertoires, including hornets^97^. Caste-enriched GO terms for the differentially expressed genes (FDR < 0.05, Supplementary Table ST32) included pheromone-related activity (GO:0016229, also upregulated in experimentally-isolated bumblebee brains^98^), as well as oxidoreductase activity (GO:0016491) involved in reprogramming cell metabolism and previous found to be caste-biased in social Hymenoptera^32,99^.

We examined whether any of the differentially expressed genes in *V. crabro* had experienced positive selection or duplication events. A single locus was found in both the dataset of duplicated genes (duplicated in OrthoFinder analysis; aim 2) and differentially expressed between castes (Vcabro1a000853P1), which shares sequence similarity with heterogeneous nuclear ribonucleoprotein H in *Apis mellifera* (Supplementary Table ST31). This locus has previously been identified as caste-biased in Cape honey bees, where it is implicated in alternative splicing leading to worker ovary activation^100^. It is also known in several bee species to recognise epigenetic modifications and is part of the post-transcriptional RNA modification machinery^101^.

## Conclusion

In this study, we sequenced, assembled and annotated two new *Vespa* genomes; we also generated gene annotation files, available for the community. We then conducted an analysis of gene evolution for vespine wasps, comparing with other social Hymenoptera. We detected high levels of gene duplications among genes associated with reproduction, communication and production of phenotypic variation in the *Vespa* species; for instance, two genes related to odorant receptors are duplicated in *V. velutina*. Furthermore, we found 104 genes under positive selection in *V. crabro*, including some associated with reproduction such as oogenesis. Finally, we provide the first analyses of caste-specific brain gene expression for a superorganismal wasp, comparing transcriptomes derived from adult workers and unmated female reproductives (gynes).

Our analyses of duplications, positive selection and differentially expressed genes detected associations with the olfactory system. Communication is behaviourally key to all social insects for reproduction^102^, colony health^103^ and species’ survival, including invasive species^104^. This is supported by evolutionary variation in chemosensory repertoire^105^ in social and invasive insects, for instance, loss of gustatory receptors in caterpillar pests, and duplicated chemosensory genes in invasive insects. Genes belonging to the zinc finger family were also found repeatedly in our analyses. This is one of the largest families of transcription factors, and so may not be so surprising. However, we suggest that this is a biologically interesting result: zinc finger domains found to be under selection in the *Vespa* lineage (duplicated, dN/dS, differentially expressed) may have experienced lineage-specific expansions and may be related to new gene regulatory functions^106,107^. Future work should focus on conducting functional tests, e.g. CRISPR to demonstrate functionality.

Accordingly, the genomic resources and analyses we provide secures the hornets a place in the ever-growing world of genome sequencing and analyses, and adds significantly to what remains an underrepresented group of insects in the genomics world—the aculeate wasps^7^. As an example of the exponential growth of genomics datasets leading to population genetics and pangenome analyses, since our analyses, the Darwin Tree of Life initiative made available a new *Vespa crabro* assembly. These new datasets will improve the scope of comparative analyses in the Hymenoptera by, for instance, providing a more complete survey of non-coding regions and olfactory receptors related to the evolution of social organisation in insects. Finally, our datasets hint at candidate genes with important gene functions—namely those involved in regulation of reproduction and communication, and open up new opportunities to explore the molecular mechanisms underpinning key ecological and physiological traits, such as those associated with invasive species and venom components in invasive *Vespula* species^76^.

Future work should focus on population genetic studies to further explore selection pressures associated with social evolution and anthropogenic impacts (e.g*.* high F_ST_ regions indicating directional selection and Tajima’s *D* indicating balancing selection within species, especially in the context of comparison between invasive and native ranges). Furthermore, it would be interesting to conduct a comprehensive TE analysis across wasps to assess potential functions related to phenotypic plasticity, for instance when comparing native and invasive species^108–110^. Finally, we encourage single-cell brain expression analyses^111^, and analysis of multiple tissues from across the diversity of phenotypes^112^ to further explore differential gene expression in *Vespa crabro*. Such approaches will help build a better understanding of these ecological and economically important insects.

## Supplementary Information

Supplementary Information 1.

Supplementary Information 2.

## Data Availability

The datasets generated during and analysed during the current study are available in the NCBI repository, SRR11213734, SRR11213735, SRR11213736, JAITYU000000000, SRX9350949, SRX9350950, SRX9350951, SRX9350952, SRX9350953, SRX9350956, SRX9350958, SRX9350959, SRX9350960, ERS3567203, PRJEB46979, SRR22217221, SRR22217222. The annotations are available on Github: https://github.com/EmelineFavreau/Vespa-Genomes-Analyses/tree/master/input/gff.
