# Supplementary material for: Putting hornets on the genomic map
Source: Sci Rep. 2023 Apr 21;13:6232. doi: 10.1038/s41598-023-31932-x (PMC10121689; doi:10.1038/s41598-023-31932-x)
Supplement: Supplementary file 2 — Supplementary Information 2. [file 41598_2023_31932_MOESM2_ESM.docx]

# Putting hornets on the genomic map

**Author list**

Emeline Favreau^1*^, Alessandro Cini^1 2^, Daisy Taylor^1^, Francisco Câmara Ferreira^3^, Michael A. Bentley^1^, Federico Cappa^4^, Rita Cervo^4^, Eyal Privman^5^, Jadesada Schneider^1^, Denis Thiéry^6^, Rahia Mashoodh^1^, Christopher D. R. Wyatt^1^, Robert L. Brown^7^, Alexandrina Bodrug-Schepers ^8^, Nancy Stralis-Pavese^8^, Juliane C. Dohm^8^, Daniel Mead^9^, Heinz Himmelbauer^8^, Roderic Guigo^3 10^, Seirian Sumner^1^

# Supplementary Information

## Materials and Methods

### A) Sequencing, Assembly and Annotation of the genome for the European hornet, *Vespa crabro*

#### DNA Extraction for *Vespa crabro*

We extracted DNA from two adult males sampled from England. We used Qiagen DNeasy blood and tissue kit (Cat. No. / ID: 69504), following the Qiagen protocol for extracting total DNA from insects using an electric homogeniser. Our protocol steps are as follows.

We first dissected brain tissue in PBS (to remove RNAlater) directly into a 2mL tube containing one steel ball and kept on liquid nitrogen to flash freeze tissue. We then transferred it immediately into –80℃ to keep frozen until required. We used between 25-50mg tissue into 180μL PBS. We homogenized using TissueLyser at 50Hz for 2-4 min. We then added 20μL Proteinase K and 200μL Buffer AL. We incubated at 56 ℃ until completely lysed (1-3 hours).

Then, we vortexed sporadically to disperse. We added 200μL Ethanol, and vortexed thoroughly. We then pipetted the mixture into a spin column in a 2mL tube. We centrifuged it at 6000 x g (8000rpm) for 1 min. We discarded the flow-through and collection tube, placed the column in a new collection tube. We added 500μL buffer AW1 centrifuge as above. We discarded flow-through and collection tube, placed the column in a new collection tube. We added 500μL buffer AW2, centrifuged for 3 mins at 20,000 g (14000 rpm). We carefully discarded the flow-through and collection tube. We then placed the column in new 1.5mL Eppendorf, added 100-200μL buffer AE. We incubated at room temperature for 1-10 min then centrifuge for 1 min at 6000 g. We repeated elution steps into a new collection tube to increase final yield.

#### Genome Sequencing and Assembly for *Vespa crabro*

Quantification of *V. crabro* genomic DNA was performed with a Qubit 3.0 fluorometer using a dsDNA BR assay kit (Thermo Fisher, Waltham, MA, USA). DNA integrity was checked on a 0.7% agarose gel. The preparation of sequencing libraries was performed using DNA isolated from haploid males. Due to the low amount of DNA available per sample, two different individuals were used for generation of a paired-end (PE; sample M1_VC1.1) and a mate-pair (MP; sample M3_VC3.1) library, respectively. A PE sequencing library with a peak insert size of 500 bp (**Figure SF1**) was constructed using 80 ng of genomic DNA of *V. crabro* sample M1_VC1.1 with a TruSeq Nano LT library preparation kit (Cat # FC-121-4001, Illumina, San Diego, CA, USA) according to the kit supplier’s instructions except that library amplification was performed with nine PCR cycles. An MP sequencing library with a peak span size of 1550 bp and a mean span size of 2432 bp (**Figure SF1**) was generated from genomic 690 ng of genomic DNA by tagmentation using a Nextera mate-pair library preparation kit (Cat # FC-132-1001, Illumina) without size selection. The MP library was amplified with 11 PCR cycles. The quality and quantity of the libraries was checked on a DNA 1000 chip on the Agilent Bioanalyzer 2100 (Agilent, Santa Clara, CA, USA). Illumina sequencing was done at the Vienna BioCenter Core Facilities (VBCF), Vienna, Austria, on a HiSeq 2500 instrument with v4 Illumina sequencing chemistry, combined with a 2x125 cycle sequencing recipe.

Raw sequencing data underwent quality control with FastQC ^1^; thereafter, trimmomatic ^2^ was employed for data filtering based on phred scores, using the following parameters: LEADING:25 TRAILING:25 SLIDINGWINDOW:10:25 MINLEN:36. We aimed to assemble contings from one haploid male, and scaffolding the resulting assembly with the other male data, thus eliminating the need for haplotype purging. We performed genome assemblies using SOAPdenovo_v2.04 ^3^. We first calculated preliminary assemblies based on PE reads ^4^, in order to assess the insert size distribution of PE reads and the span size distribution of MP reads, respectively, by mapping onto such pre-assemblies. We used bowtie2 ^5^ with an insert size interval between 100 nt and 1200 nt (bowtie2 --fr -I 100 -X 1200 -1 pe_reads1.fastq -2 pe_reads2.fastq -x preassembly_1) and sampled two million PE read-pairs to estimate the library insert size (**Figure SF1a**). To estimate the span size of the MP read-pairs, we used bowtie2 and an insert size interval between 100 nt and 20000 nt (bowtie2 --rf -I 100 -X 20000 -1 mp_reads1.fastq -2 mp_reads2.fastq -x preassembly_2), and sampled two million MP read-pairs (**Figure SF1b**). Using the determined library insert size and MP span size as parameters for the assembly run, several assemblies were calculated from the quality-filtered sequencing reads by varying the k-mer size parameter between 23 and 125. An assembly calculated with k-mer size 91 (SOAPdenovo-63mer all -s assembly.config -K 91 -R -o assembly.K91) was the best performing in terms of core gene presence despite not having the best N50 as assessed using QUAST ^6^. BUSCOv3 ^7,8^ was used in genome mode with the hymenoptera_odb9 lineage and honeybee1 species to assess assembly gene completeness (blast 2.2.30, AUGUSTUS 3.2.1). We submitted the assembly to NCBI. A contamination screen is performed by default by NCBI when submitting a genome assembly, and a list of suspicious contigs is returned to the submitter. Assemblies are only provided with accession numbers once suspicious sequences have been removed. Hence, we can consider the *V. crabro* assembly as clean. Metrics of the final assembly were determined with custom scripts ^4^, taking only sequences larger than 500 bp into account. Jellyfish 2.2.10 ^9^ was used to determine genome size based on the quality-filtered Illumina PE sequencing reads. Bioawk was used to retrieve the GC content of all the reads as well as non-overlapping 125 nt segments of the final assembly lacking undetermined bases (no unknown nucleotide “N”). Calculations were performed on a cluster node with 32 cores of 3.3 GHz and 128 GB memory. Plotting of data was done using R version 3.2.2 ^10^.

#### Structural Annotation of *Vespa crabro* Genome

##### 1.1 Repeat masking of the *V. crabro* genome assembly

The genome assembly was first modified by removing all contigs with fewer than 500 bases as these were previously determined to most likely not to include any complete genes using the genome assembly "gene space" assessment program BUSCOv2 ^7^. Then we executed RepeatMasker (v4.0.5) using Hymenoptera repeat libraries in order to find and mask transposable elements as these reduce the accuracy of gene prediction tools. We used RepeatMasker through the repeat masking module of the genome annotation program Maker v2.31.9 ^11^ which also uses RepeatRunner to further increase the accuracy of transposon finding. We were able to transposon-repeat mask 4.78% of the assembly made up of more than 500 bases.

##### 1.2 Generation of genome-guided RNASeq alignments

We aligned Illumina paired-end RNASeq data representative of *V. crabro* tissues to the assembly of the *V. crabro* genome using the STAR RNASeq aligner v2.5.1b ^12^ using the program's default parameters for paired-end RNASeq data.

##### 1.3 Transcript assembly reconstruction of RNASeq alignments

We proceeded to assemble the *V. crabro* RNASeq alignments generated by the STAR aligner into potential genes/transcripts by using the transcriptome reconstruction programs StringTie v1.3.3b ^13^, CuffLinks v2.2.1 ^14^, CLASS v2.17 ^15^ and Scripture beta2 ^16^ using the default parameters (for paired-end data when available) for each of these tools. The output of each of these tools was then combined and passed to the PASA (Program to Assemble Spliced Alignments, v2.0.2) pipeline ^17^.

##### 1.4. Generation of a PASA-derived transcriptome

The PASA pipeline was used to combine the output of the transcript assembly programs in section 1.3 into a more exhaustive transcriptome as well as to generate a TransDecoder-derived ^18^ *ab initio* program training set employed to train the ab initio programs used by the pipeline. We also provided PASA with all the 40,177 RefSeq protein-coding transcripts sequences from all organisms classified as "wasps" found in the NCBI nucleotide database (https://www.ncbi.nlm.nih.gov/nuccore). In total we provided a PASA pipeline (which uses GMAP/BLAT as the alignment engines) with 297,206 transcripts which were processed into 82,198 maximal PASA transcript assemblies. PASA is set to be quite stringent; input sequences with less than 95% identity to the genomic sequence over 95% of their length were discarded. Furthermore, PASA generates maximal assembly of spliced alignments (the transcript alignments deemed as valid are clustered based on genome mapping location and assembled into gene structures that include the maximal number of compatible transcript alignments).

##### 1.5. Obtaining protein-coding *ab initio*/evidence-based gene predictions

###### 1.5.1. Geneid (geneid+introns) V. crabro-specific gene predictions

Geneid ^19^ is an *ab initio* gene prediction program used to find potential protein-coding genes in anonymous genomic sequences. In the context of geneid, training basically consists of computing position weight matrices (PWMs) or Markov models of order 1 for splice sites and start codons, and deriving a model of coding DNA (generally a Markov model of order 5). Furthermore, once a preliminary species-specific matrix is obtained it is further optimized by adjusting two internal matrix parameters: the cutoff of the scores of the predicted exons (eWF) and the ratio of signal to coding statistics information to be used (oWF).

The initial training set for *V. crabro* was generated using one of the modules of the PASA pipeline and comprised 2,459 gene models randomly selected from within the set of 7,000 longest ORFs (more than 150 amino-acids) corresponding to complete/non-overlapping genes produced by PASA (see sections 1.4 and 1.6 for more information). These training-set genes were also selected because they spanned over 98% of the full-length of the annotated protein sequences of the NCBI non-redundant (NR) database (using the algorithm BLASTP, E=10^-3^, minimum identity=25%; NR database version of Feb. 2017). Of these gene models 80% (1,967) were used to train geneid (and most of the other *ab initio* tools used in this study) while the remaining 20% (492) were set aside to test the accuracy of the newly developed matrices. The 1,967 *V. crabro* training-set protein-coding gene models included 11,306 canonical donor splice sites/11,407 canonical acceptor sites and 1,967 start codons. These start codons were used to compute PWMs while the donor and acceptors were employed to derive Markov matrices of order 1. We also had enough coding/non-coding nucleotides to derive a Markov of order 5 as a model for the coding potential. Accuracy of the geneid parameter file was tested on an evaluation “artificial scaffold”, consisting of the 492 evaluation-set concatenated gene models with 800 nucleotides of intervening sequence between each of the genes (**Table ST3 A**) and subsequently used to generate genome-wide predictions.

Geneid can also use external information, such as the coordinates of known introns, to improve the accuracy of its predictions. In order to take advantage of this feature of geneid we first extracted and scored all potential introns from the "spliced junctions" (SJ) file generated by the STAR RNASeq alignment tool. This resulted in a set of 141,233 introns, of which we selected 92,027 on the basis that they overlapped with geneid predictions. To measure the accuracy of the geneid gene predictions when using intronic evidence we used STAR on the artificial scaffold to generate RNASeq alignments and then used the output SJ file to obtain all the potential intron sequences. The introns not overlapping with geneid predictions were then filtered out. Subsequently, we calculated the accuracy of geneid (+introns) on the test scaffold (excluding the mono-exonic genes), which showed an improvement in the performance of the program when using introns as evidence (**Table ST3 A**). The parameter file (and genome-wide intron data) were then used to obtain predictions on the entire genome assembly.

The training of geneid to obtain a parameter file for *V. crabro* was based on the method described to obtain a *Drosophila melanogaster* geneid parameter file ^20^. Training was performed in a “semi-automated“ fashion by employing an in–house geneid training tool (geneidTRAINer1.1).

###### 1.5.2 GlimmerHMM V. crabro-specific gene predictions.

We also obtained *V. crabro*-specific matrices for the gene prediction tool glimmerHMM ^21^. GlimmerHMM is an *ab initio* program that is based on a Generalized Hidden Markov Model (GHMM). This program also incorporates splice site models adapted from the GeneSplicer program and a decision tree adapted from glimmerM. We generated the species-specific matrix for this program by using a “self-training” script obtained from the University of Maryland (http://www.cbcb.umd.edu/software/GlimmerHMM). The training was performed by following the instructions provided in http://www.cbcb.umd.edu/software/GlimmerHMM/man.shtml#training. GlimmerHMM was trained on the same set of sequences used to train the gene prediction program geneid. The new glimmerHMM matrix was evaluated on the same “artificial scaffold” used to evaluate the other *ab initio* gene prediction parameter files described in this text (results are shown in **Table ST3 A**) and subsequently used to generate glimmerHMM genome-wide predictions.

###### 1.5.3 GeneMarkES and geneMarkET V. crabro-specific gene predictions

We generated an *V. crabro*-specific matrix for the gene prediction program geneMark ^22^. The GeneMark-ES and GeneMark-ET algorithms were developed for finding protein-coding genes in eukaryotic genomes without training sets. GeneMark-ES determines species-specific gene finding parameters using a self-training algorithm based on the species of interest genomic sequence. GeneMark-ET does the same as GeneMark-ES but it can also use intron data to improve the accuracy of the predictions. We generated an *V. crabro*-specific matrices for this program by using the training sequence fastas generated by GeneidTrainer and following the self-training instructions found both at the program developer’s website (http://opal.biology.gatech.edu/) and as indicated by Lomsadze et al. ^22^. GeneMark-ES/ET were trained on the artificial training scaffold generated by GeneidTRAINer1.1 and geneMark-ET also used the introns (overlapping with geneid predictions) derived from the SJ file generated by STAR after aligning all-tissue RNASeq data on the artificial training scaffold. The new geneMark-ES and geneMark-ET matrices were evaluated on the same artificial scaffold used to evaluate other *ab initio* gene prediction parameter files used in this study (**Table ST3 A**). Predictions were subsequently obtained by running the programs on the entire genome of *V. crabro*.

###### 1.5.4 Augustus V. crabro-specific gene predictions

We also built an *V. crabro*-specific parameter file for the gene prediction program Augustus ^23^. Augustus is a program that predicts genes in eukaryotic genomic sequences and that is also “re-trainable”. The program is based on a Hidden Markov Model and integrates a number of known methods and sub-models. In order to obtain parameter files for Augustus we employed its own training program (http://www.molecularevolution.org/molevolfiles/exercises/augustus/training.html), and used it to estimate the optimal parameters for *V. crabro* given the same 1,967 species-specific genes used in training the other *ab initio* tools used to obtain gene predictions on these species. The resulting Augustus parameter file was evaluated on the same “artificial scaffold” consisting of the 492 concatenated gene models with 800 nucleotides of intervening sequence used to evaluate the other programs previously described (**Table ST3 A**), and subsequently used to generate genome-wide gene predictions.

We also took advantage of Augustus’ potential to use external evidence to improve its performance. We did this by obtaining a set of predictions that used the newly developed *V. crabro* Augustus parameter file in combination with PASA-derived transcript evidence obtained for this species (refer to section 1.2 for additional information). Our strategy for taking advantage of the large set of transcripts followed the methodology described in (http://Augustus.gobics.de/binaries/readme.rnaseq.html) and in an article by Stanke et al. ^23^ and allowed us to obtain a higher-accuracy evidence-based set of Augustus predictions on the *V. crabro* assemblies (refer to **Table ST3 A**). In order for the *V. crabro* Augustus matrix to take advantage of the external data we first had to optimize some internal parameters of the new Augustus parameter file; the exonpart bonus for hints corresponding to PASA-evidence (“E”) was given a bonus of 1xE^3^. Also, for every exonpart that was not supported by the PASA evidence, the probability of the gene structure was given a “malus” or a penalization of 0.997. Furthermore, complete exons predicted by Augustus that perfectly matched the exons in the external hints were given a bonus of 1xE^4^. The intron bonus for (PASA) hints of source E was set to 1xE^5^, meaning that a predicted intron would get this bonus when being exactly as in the PASA “hint”. The Augustus parameter file plus the "hints" evidence file derived from the PASA transcriptome was also tested against the artificial scaffold as before and used to predict genes on the whole genome assembly.

###### 1.5.5 SNAP V. crabro-specific gene predictions

Our final source of *ab initio* gene predictions to be used by the EVM combiner was obtained using the program SNAP ^24^ using a *V. crabro*-specific matrix after training the program using a suite of self-training scripts included within the package. SNAP was developed by Ian Korf and consists of a general-purpose gene finding program that can be used both on eukaryotic and prokaryotic genomes. SNAP is an acronym for “Semi-HMM-based Nucleic Aid Parser”. SNAP was trained on the same set of sequences used to train the gene prediction program geneid. The new glimmerHMM matrix was evaluated on the same “artificial scaffold” used to evaluate the other *ab initio* gene prediction parameter files described in this text (results are shown in **Table ST3 A**) and subsequently used to generate SNAP genome-wide predictions.

###### 1.5.6 Combining gene prediction data of different gene prediction programs

Geneid (with or without introns), Augustus (with or without “hints”), glimmerHMM, geneMarkES, geneMarkET and SNAP, using their newly developed *V. crabro* -specific parameter files were subsequently used to predict genes on the repeat-masked assembly of this genome (V.crabro.RM.hymenoptera.fasta). The current *V. crabro* assembly is made up of 30,304 scaffolds/contigs with more than 500 bases. Given the species-specific parameter file developed for the organism in this study, geneid predicted 44,476 protein-coding genes without external evidence and 10,348 sequences when using intronic data. The *ab initio* tool glimmerHMM produced 32,105 gene models on the scaffolds of *V. crabro*. The program Augustus predicted 17,557 genes on the assembly while its evidence-based variation of Augustus(+hints) produced 19,415 predictions. The programs GeneMark-ES generated 16,100 gene models whereas GeneMark-ET (using intron evidence) produced 15,320 gene predictions. SNAP generated 38,068 predictions on the assembly of *V. crabro*. The full set of TransDecoder-derived gene models generated by the training-set module of the PASA pipeline (section 1.4) and the output of the programs above were used as input to a “combiner” (Evidence Modeler; EVM r2012-06-25) ^25^), which was the program employed to obtain the reference annotation for this genome.

##### 1.6 EVM-based genome annotation of the *V. crabro* assembly by combining different sources of evidence using weights.

A combination of the Program to Assemble Spliced Alignments (PASA v2.0.2) ^17^ and Evidence Modeler (EVM r2012-06-25) ^25^ were used to obtain consensus coding sequence (CDS) models using three main sources of evidence: aligned transcripts (sections 1.3-1.4), aligned proteins, and gene predictions (section 1.5).

###### 1.6.1 PASA transcript alignments

The *V. crabro* RNA sequences processed by the PASA pipeline (v2.0.2) were obtained as briefly described in section 1.4. This process resulted in PASA transcript assemblies. The transcriptome was subsequently added to the PASA database.

###### 1.6.2 Protein alignments

In order to generate protein-alignment data for EVM all 17,084 model wasp *N. vitripennis* UniProt-derived protein sequences (Feb 2017), highly curated 26,634 invertebrate SwissProt proteins (Feb 2017) and NCBI protein-coding RefSeqs (40,177 sequences; Feb 2017) classified as belonging to "wasps" were split-mapped to the *V. crabro* genome by using the program SPALN2 ^26^ with hymenoptera-specific parameters.

Furthermore, we also used the spliced-protein alignment tool exonerate ^27^ to map the invertebrate SwissProt protein sequences to the scaffolds of *V. crabro* (**Table ST3 B**).

###### 1.6.3 Combining the different EVM sources

The resulting alignments were then filtered as suggested in the EVM documentation (https://evidencemodeler.github.io/). Gene predictions were obtained as previously described (sections 1.5.1 - 1.5.6) and also modified as recommended (https://evidencemodeler.github.io/) and added to the EVM pipeline. We also used TransDecoder annotations generated by the PASA training-set generation module that were classified as "other predictions" in the EVM weights file. Subsequently the transcript alignments, protein alignments and the *ab initio* gene models were combined into consensus CDS models by EVM using different weights. The best combination of weights (shown in **Table ST3 B**) were selected following the instructions contained within the EVM documentation and by previously running a "mock" EVM annotations, using a wide range of different weight files, and selecting the weights file that produced the most accurate test EVM annotation on the same “artificial scaffold” consisting of the 281 concatenated gene models with 800 nucleotides of intervening sequence previously used to evaluate all *ab initio* programs. Furthermore, with regard to the *ab initio* predictions, the weights given to each of the tools was based on the accuracy of the different programs in predicting sequences on the evaluation “artificial scaffold” for this species (refer to section 1.5.1 and **Table ST3 A**).

We also determined which sources of evidence (*ab initio*, protein or transcript) EVM used to build each of the 16,486 consensus gene models (Refer to **Table ST3 C**). The consensus CDS models were then updated with UTRs and alternative exons through four rounds of PASA’s routine to update annotations. The resulting 21,285 transcripts were grouped into (16,409) genes, transcripts and (19,597) proteins and then a pre-selected species-specific identifier was assigned to the genes, transcripts and protein products derived from them.

We then proceeded to add the sources of evidence EVM used to build each of the 16,409 gene models to the “gene” rows of the reference gff3 file produced by this project. The identifiers corresponding to the different source of evidence are as follows: trans_ev (PASA or TRANSDECODER transcript-derived evidence), novel_trans_ev (PASA or TRANSDECODER evidence obtained after EVM is run through PASA updates; no *ab initio* or protein support), prot_ev (Protein evidence from any of the four sources of spliced-protein alignments provided to EVM for this species), geneid (geneid *ab initio* predictions), geneid+i (geneid predictions using external intron evidence), Augustus (Augustus *ab initio* predictions), Augustushints (Augustus predictions using evidence derived from the PASA transcripts), genemark (genemarkES *ab initio* or genemarkET evidence-based predictions), GLIMMERHMM (glimmerHMM ab initio predictions), SNAP (snap *ab initio* predictions).

###### 1.6.4 EVM consensus annotation statistics

Finally, and as a quality control, the protein products obtained from the reference annotation of this species was aligned against either the exhaustive NCBI non-redundant (NR-201701) database using the “protein vs. protein” BLASTP “flavor” of the sequence comparison tool BLAST (E=10^-2^ with a minimum identity of 25%) to determine what percentage of the annotated genes matched a sequence of this large biological-sequence public databases. Results showed that 78.7% of our consensus EVM reference of this species matched an NR protein given the criteria above (**Table ST3 C**). Furthermore, **Table ST3 D** contains a wide-range of statistics obtained from the *V. crabro* assembly and analysis of the consensus EVM protein-coding reference annotation set obtained for this species.

### **B)** Sequencing, Assembly and Annotation of the genome for the yellow-legged Asian hornet, *Vespa velutina*

#### DNA extraction for *Vespa velutina*

We used DNA from one female worker head and one adult male sampled from Italy and stored in ethanol. DNA was extracted at the Wellcome Sanger Institute Scientific Operations core. The iyVesVel sample was weighed and dissected on dry ice. Thorax tissue was disrupted manually using a sterile plastic pestle. High molecular weight (HMW) DNA was extracted using the Qiagen MagAttract HMW DNA extraction kit. Fragment size analysis of 0.25ng of DNA was then performed using an Agilent FemtoPulse.

#### RNA extraction for *Vespa velutina*

We sampled gynes and queens into RNALater from nests in Villenave D'Ornon and Vayres (France). We extracted RNA using published protocols (Taylor al 2020) and RNeasy Mini Kit (Qiagen). Vvel_Q is a sample made of one queen (nest 2), and Vvel_G4 is a sample made of five gynes (nests 1, 3, 4 and 5). Novogene Co ran QC (Nanodrop and TapeStation), then produced Illumina libraries (pair-end kit) before sequencing on Illumina HiSeq 2000, aiming for150bp paired-end reads to a depth of 10 million reads/sample. These samples are used as guidance in genome annotation.

#### Genome Sequencing and Assembly for *Vespa velutina*

For the PacBio sequencing, we first needle-sheared the sample, then used the PacBio Express Library kit. Size selection was performed with Blue Pippin (15kb cut). The 10x Genomics Chromium library and a Hi-C library were produced using manufacturers’ instructions. All three libraries were subsequently sequenced at the Wellcome Sanger Institute (respectively on Sequel platform for 56x coverage and on Illumina HiSeq X platform for 94x coverage); and at the Baylor College of Medicine (Illumina NovaSeq 6000 platform). We assembled the genome in several steps. Briefly, we ran a palindromic read correction with pbclip (https://github.com/fenderglass/pbclip) and generated an initial PacBio assembly with Falcon-unzip ^28^ and we retained haplotig separation with purge_dups (https://github.com/dfguan/purge_dups). We scaffolded the assembly with Hi-C data using SALSA2 ^29^; we polished with Arrow (https://github.com/PacificBiosciences/GenomicConsensus), and FreeBayes-called variants ^30^ from 10X Genomics Chromium reads aligned with LongRanger. Finally, the assembly was analysed, manually improved using gEVAL ^31^ and submitted (NCBI accession GCA_912470025.1).

#### 2. Structural Annotation for *Vespa velutina*

##### 2.1. Repeat masking of the *V. velutina* genome assembly

The method is the same as *V. crabro* (section 1). We were able to transposon-repeat mask 5.98% of the assembly (V.velutina.RM.hymenoptera.fasta).

##### 2.2. Generation of genome-guided RNASeq alignments

We aligned Illumina paired-end RNASeq data representative of a wide-range of *V. velutina* brain-derived tissues (Vvel_G4, Vvel_Q) to the assembly of the *V. crabro* genome using the STAR RNASeq aligner v2.5.1b using the program's default parameters for paired-end RNASeq data.

##### 2.3. Transcript assembly reconstruction of RNASeq alignments

The method is the same as *V. crabro* (section 1.3).

##### 2.4. Generation of a PASA-derived transcriptome

The method is the same as *V. crabro* (section 1.4) with the following exception. In total we provided a PASA pipeline (which uses GMAP/BLAT as the alignment engines) with 288,404 transcripts which it processed into 66,194 maximal PASA transcript assemblies.

##### 2.5. Obtaining protein-coding ab initio/evidence-based gene predictions

###### 2.5.1 Geneid (geneid + introns) V. velutina-specific gene predictions

The method is the same as *V. crabro* (section 1.5.1) with the following exception.The initial training set for *V. velutina* was generated using one of the modules of the PASA pipeline and comprised 1,972 gene models randomly selected from within the set of 9,530 longest ORFs (more than 150 amino-acids) corresponding to complete/non-overlapping genes produced by PASA (see sections 2.4 and 2.6 for more information). Of these gene models 80% (1,577) were used to train geneid (and most of the other *ab initio* tools used in this study) while the remaining 20% (395) were set aside to test the accuracy of the newly developed matrices. The 1,577 *V. velutina* training-set protein-coding gene models included 11,468 canonical donor splice sites/11,627 canonical acceptor sites and 1,554 start codons. Accuracy of the geneid parameter file was tested on an evaluation “artificial scaffold”, consisting of the 395 evaluation-set concatenated gene models with 800 nucleotides of intervening sequence between each of the genes (**Table ST3 A**) and subsequently used to generate genome-wide predictions. Scoring introns from spliced junctions file resulted in a set of 246,963 introns, of which we selected 175,349 on the basis that they overlapped with geneid predictions.

###### 2.5.2 GlimmerHMM V. velutina-specific gene predictions

The method is the same as *V. crabro* (section 1.5.2).

###### 2.5.3 GeneMarkES and geneMarkET V. velutina-specific gene predictions

The method is the same as *V. crabro* (section 1.5.3).

###### 2.5.4 Augustus V. velutina-specific gene predictions

The method is the same as *V. crabro* (section 1.5.4), with the following exception. The resulting Augustus parameter file was evaluated on the same “artificial scaffold” consisting of the 395 concatenated gene models with 800 nucleotides of intervening sequence used to evaluate the other programs previously described.

###### 2.5.5 SNAP V. velutina-specific gene predictions

The method is the same as *V. crabro* (section 1.5.5).

###### 2.5.6 Combining gene prediction data of different gene prediction programs

The method is the same as *V. crabro* (section 1.5.6), with the following exception. The current *V. velutina* assembly is made up of 43 scaffolds. Given the species-specific parameter file developed for the organism in this study, geneid predicted 17,152 protein-coding genes without external evidence and 9,961 sequences when using intronic data. The *ab initio* tool glimmerHMM produced 25,241 gene models on the scaffolds of *V. velutina*. The program Augustus predicted 15,020 genes on the assembly while its evidence-based variation of Augustus(+hints) produced 13,400 predictions. The programs GeneMark-ES generated 17,852 gene models whereas GeneMark-ET (using intron evidence) produced 22,979 gene predictions. SNAP generated 37,500 predictions on the assembly of *V. velutina*.

##### 2.6 EVM-based genome annotation of the V. *velutina* assembly by combining different sources of evidence using weights.

The method is the same as *V. crabro* (section 1.6).

###### 2.6.1 PASA transcript alignments

The method is the same as *V. crabro* (section 1.6.1).

###### 2.6.2 Protein alignments

The method is the same as *V. crabro* (section 1.6.2).

###### 2.6.3 Combining the different EVM sources

The method is the same as *V. crabro* (section 1.6.3), with the following exception. We used 395 concatenated gene models. We also determined which sources of evidence (*ab initio*, protein or transcript) EVM used to build each of the 13,200 consensus gene models (Refer to **Table ST3 C**). The consensus CDS models were then updated with UTRs and alternative exons through four rounds of PASA’s routine to update annotations. The resulting 20,107 transcripts were grouped into (12,928) genes, transcripts and (17,334) proteins and then a pre-selected species-specific identifier was assigned to the genes, transcripts and protein products derived from them.

###### 2.6.4 EVM consensus annotation statistics

The method is the same as *Vespa crabro* (section 1.6.4), with the following exception. Results showed that 87.3% of our consensus EVM reference of this species matched an NR protein given the criteria above (**Table ST3 C)**.

### C) Functional Annotation of two hornet genomes

#### Functional Annotation for *Vespa crabro*

For the functional annotation we used InterPro ^32^, PANNZER2 ^33^, Blast2GO ^34^, signalP ^35^, and NCBI CDsearch ^36^ databases. InterProScan v.5.19-58 ^37^ was used to scan through all available InterPro databases, including PANTHER, Pfam, TIGRFAM, HAMAP and SUPERFAMILY. BLASTP v.2.2.29+ search against NCBI non-redundant (NR) collection of protein sequences (release 2017-06) was used as input to the local software p2gpipe version 2.5.0, database update 2017-01. PANNZER2 (Protein ANNotation with Z-scoRE) is an automated service for functional annotation of prokaryotic and eukaryotic proteins of unknown function. The tool is designed to predict the functional description (DE) (**Table ST3 E**) and GO terms (**Table ST3 G**). We implemented a command-line remote access method for PANNZER2 in order to bypass the web-page input mode and automate the analysis as much as possible.

#### Functional Annotation for *Vespa velutina*

For the functional annotation we used InterProScan, PANNZER2, Blast Annotator using UniProt-GOA ^38^, signalP and TargetP, and NCBI CDsearch databases. InterProScan v.5.32-71 was used to scan through all available InterPro databases, including PANTHER, Pfam, TIGRFAM, HAMAP and SUPERFAMILY. BLASTP v.2.7.1+ was used to search against the NCBI non-redundant (NR) collection of protein sequences (release 2018-10). We used a command-line remote access method for PANNZER2 in order to bypass the web-page input mode and automate the analysis as much as possible. KEGG orthology (KO) groups were assigned by KEGG Automatic Annotation Server (KAAS) ^39^ using bi-directional best hit (BBH) method against a representative gene set from 28 different species, including several flukes. KO identifiers were then used to retrieve relevant functional annotation (DE using the KEGG ^40^ REST-based API service (KEGG release v89.1)).

The level of fragmentation, N50, describes a state far away from the expected number of chromosomes based on karyotype analysis (n = 25 for *Vespa crabro* and *Vespa mandarinia* ^41,42^).

## Results

### Functional Annotation

For *V. crabro*, a total of 17,677 (90.2%) out of 19,597 proteins had some type of annotation feature derived from one of the annotation resources used in this work. GO terms were assigned to 12,853 (65.59%) proteins using InterPro, PANNZER and blast_annotator (BLAST2GO) (**Table ST3 E**). Additionally, we were capable of assigning a functional description (DE) to 15,267 proteins using Blast best hit or PANNZER2 DE terms.

For *V. velutina*, a total of 16,779 (96.8%) out of 17,334 proteins had some type of annotation feature derived from one of the annotation resources used in this work. GO terms were assigned to 14,198 (81.90%) proteins using InterPro, KEGG, PANNZER and blast_annotator (BLAST2GO equivalent) (**Table ST3 E**). Additionally, we were capable of assigning a functional description (DE) to 15,079 (87.00%) proteins using Blast best hit, KEGG or PANNZER2 DE terms.

In order to functionally annotate this proteome we used InterProScan and Batch CD-search software to assign domains and other functional elements to the proteins of interest. InterProScan v.5.19-58 was used to inspect proteins for signatures using all available InterPro databases and scanning applications. For *V. crabro*, in total, 17,411 (88.84%) proteins have some type of protein signatures. More specifically, 12,845 proteins (65.55%) are annotated with at least one signature coming from one of the most important InterPro databases for functional annotation (i.e. PANTHER, Pfam, TIGRFAM, HAMAP, SUPERFAMILY). For *V. velutina*, in total, 16,003 (92.32%) proteins have some type of protein signatures. More specifically, 13,501 proteins (77.89%) are annotated with at least one signature. **Table ST3 F** displays the number of proteins containing a signature belonging to each specific InterPro member database.

Automatic Batch CD-server was used to scan a set of pre-calculated position-specific scoring matrices with proteins. For *V. crabro*, in total, 11,786 proteins have domain hits and 5,718 proteins have features data, such as active sites, inter-domain contacts, cleavage sites or proline interaction residues. For *V. velutina*, in total, 6,222 proteins have domain hits and 1,421 proteins have features data.

For *V. crabro*, we used three different sources of evidence to associate GO terms to our proteins: InterPro, PANNZER2 and blast_annotator (blast2go); for *V. velutina* four different sources: InterPro, KEGG, PANNZER2 and blast_annotator (i.e. Uniprot-GOA) (**Table ST3 G**). For *V. crabro*, in total we managed to associate at least one GO term to 12,853 proteins using all programs; with 1-22 GO terms per protein (blast_annotator) and 1-30 GO terms per protein (PANNZER2). For *Vespa velutina*, in total we managed to associate at least one GO term to 14,198 proteins using all programs; with 1-423 GO terms per protein (blast_annotator) and 1-548 GO terms per protein (PANNZER2). **Table ST3 H** displays the number of GO terms of each specific type obtained in this work.

We annotated proteins as putative transposons by using annotation signatures that were previously associated to the TEs activity. Within these signatures are the PFAM domains. We searched for around 80 transposase domains; GO terms GO:0006278 (RNA-dependent DNA replication), GO:0015074 (DNA integration), GO:0006355 (regulation of transcription, DNA-templated), GO:0004803 (transposase activity). We then searched for 'retrotransposon/transposase' within definition obtained from the Blast2GO or PANNZER2. In total we annotated 825 unique *V. crabro* proteins (622 genes) and 732 unique *V. velutina* proteins (504 genes) as putative transposons (**Table ST3 I**).

# Supplementary Figures


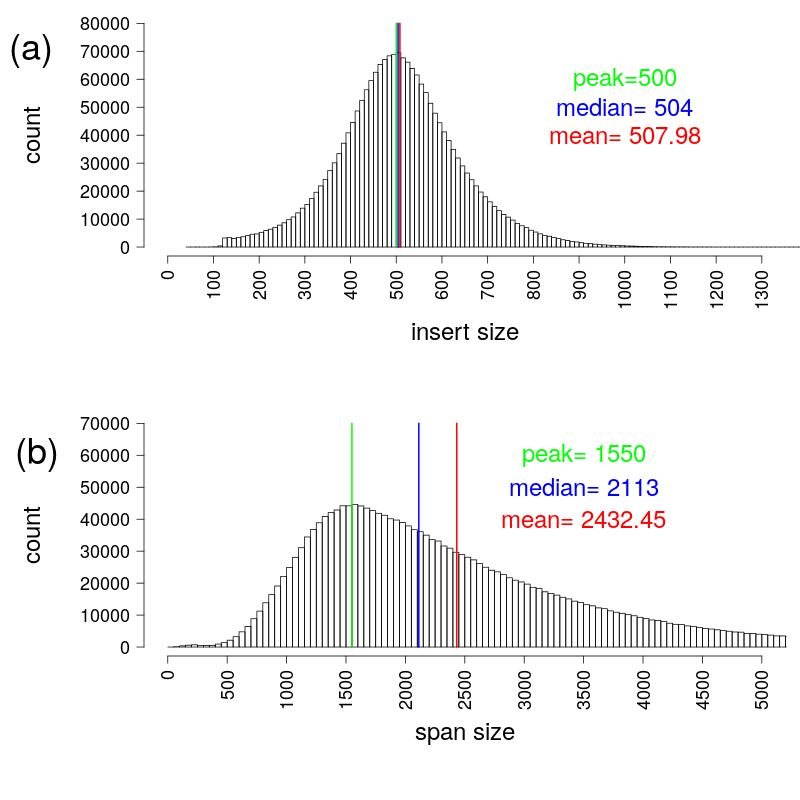


#### Figure SF1: Insert size and span size distribution of *Vespa crabro* sequencing libraries.

(a) Insert size distribution of the genomic paired-end sequencing library obtained by read mapping. (b) Span size distribution of the mate-pair library, as assessed by read mapping.


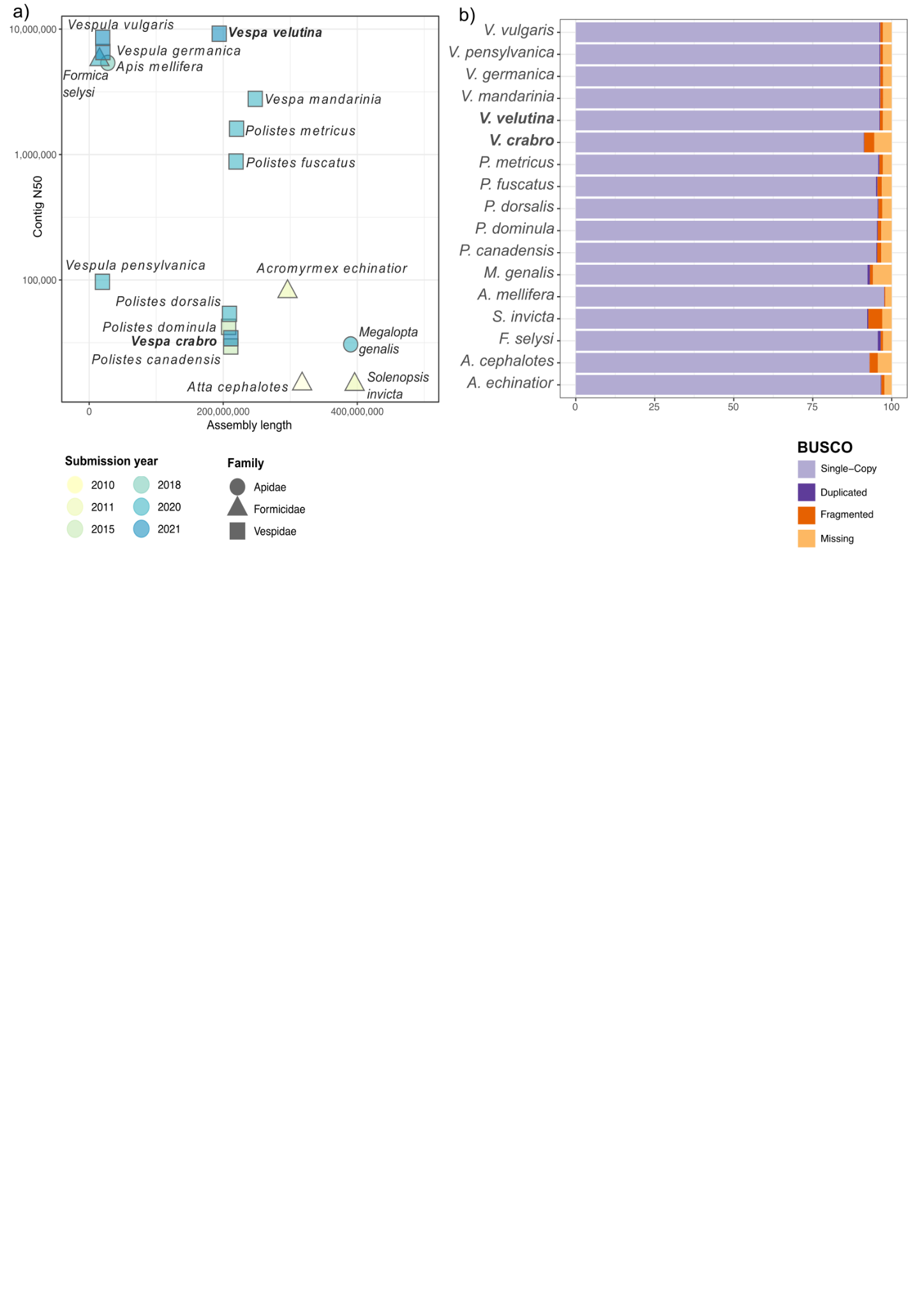


#### Figure SF2: Genome statistics for the two new genomes in comparison with other Hymenoptera genomes

**a)** Assembly length and fragmentation (contig N50) for eleven wasps, two bees, four ants.

All data in Supplementary Table ST4.


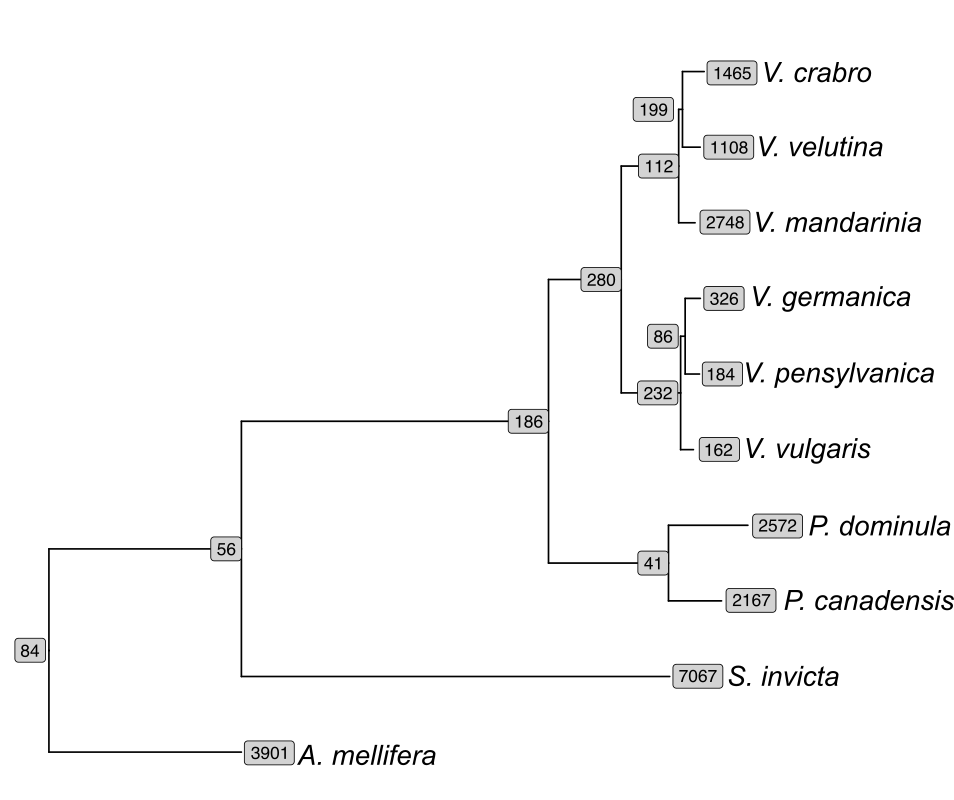


#### Figure SF3: Gene duplication events at nodes and terminal branches compared across the Hymenoptera (with a 50% support from orthoFinder).

There are 22,976 duplication events in 16,913 orthogroups, including 1,465 on the species branch of *V. crabro* and 1,108 on *V. velutina*.


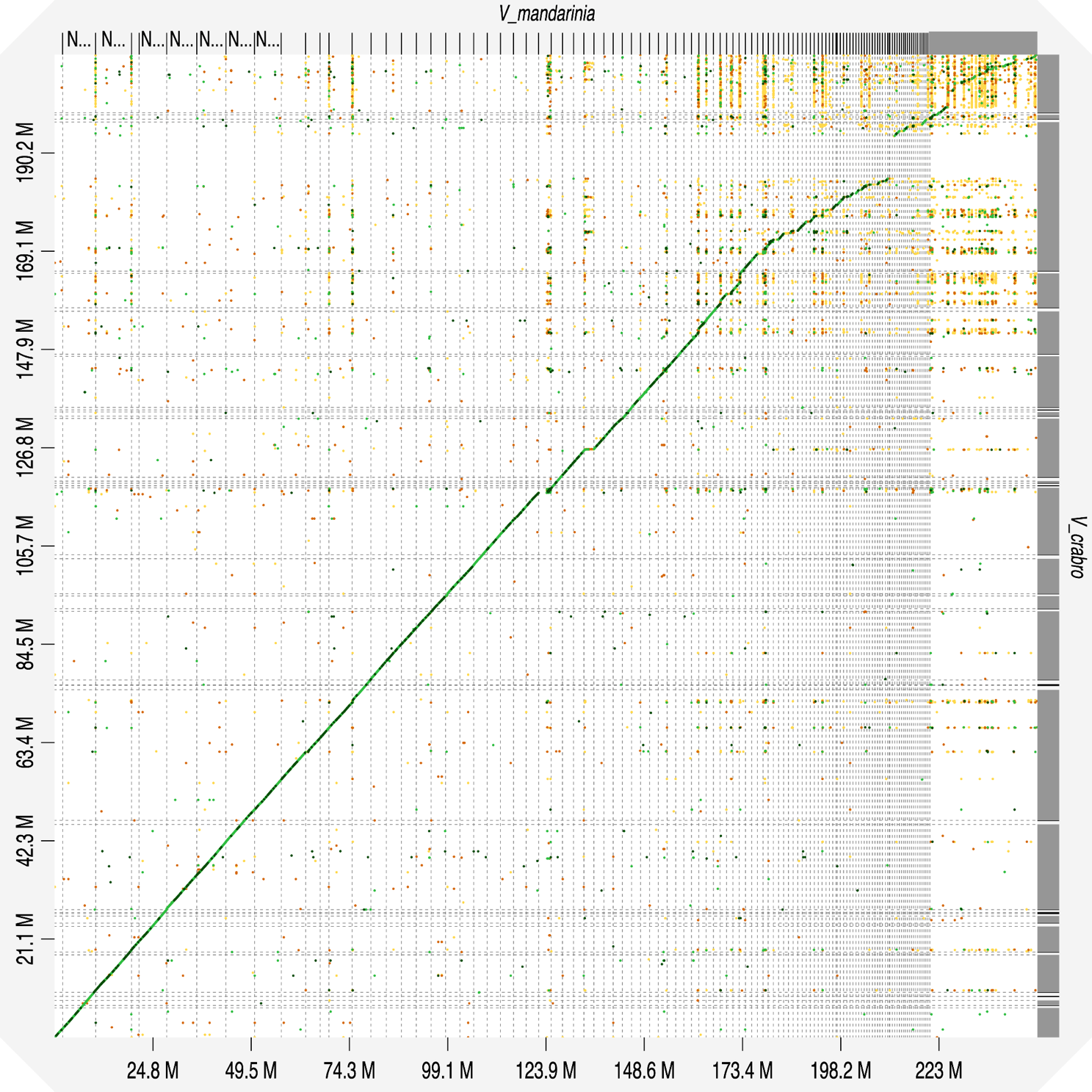


#### Figure SF4: Synteny dot plot comparing *Vespa mandarinia* and *Vespa crabro*.

DGenies dot plot from minimap2 alignment. *Vespa mandarinia* contigs are on the top (the reference), *V. crabro* contigs are on the right (the query). *V. crabro* contigs are sorted in a tailored order described as follow: For each *V. crabro* contig, DGenies searched the region which have the biggest matches with the *V. mandarinia* target and store these coordinates. *V. crabro* contigs are then by their associated coordinates. Additionally, all sequences smaller than 0.2 percent of the total length are merged in a unique super-sequence for which the margin is grayed. Each dot represents how similar the two sequences are, based on BLAST-like identity score. Each match is graded from dark green (0.75 to 1, i.e. matching very well), to light green (0.5 to 0.75, i.e. matching well), to orange (0.25 to 0.5, i.e. matching not well), to yellow (0 to 0.25, i.e. matching not well at all).


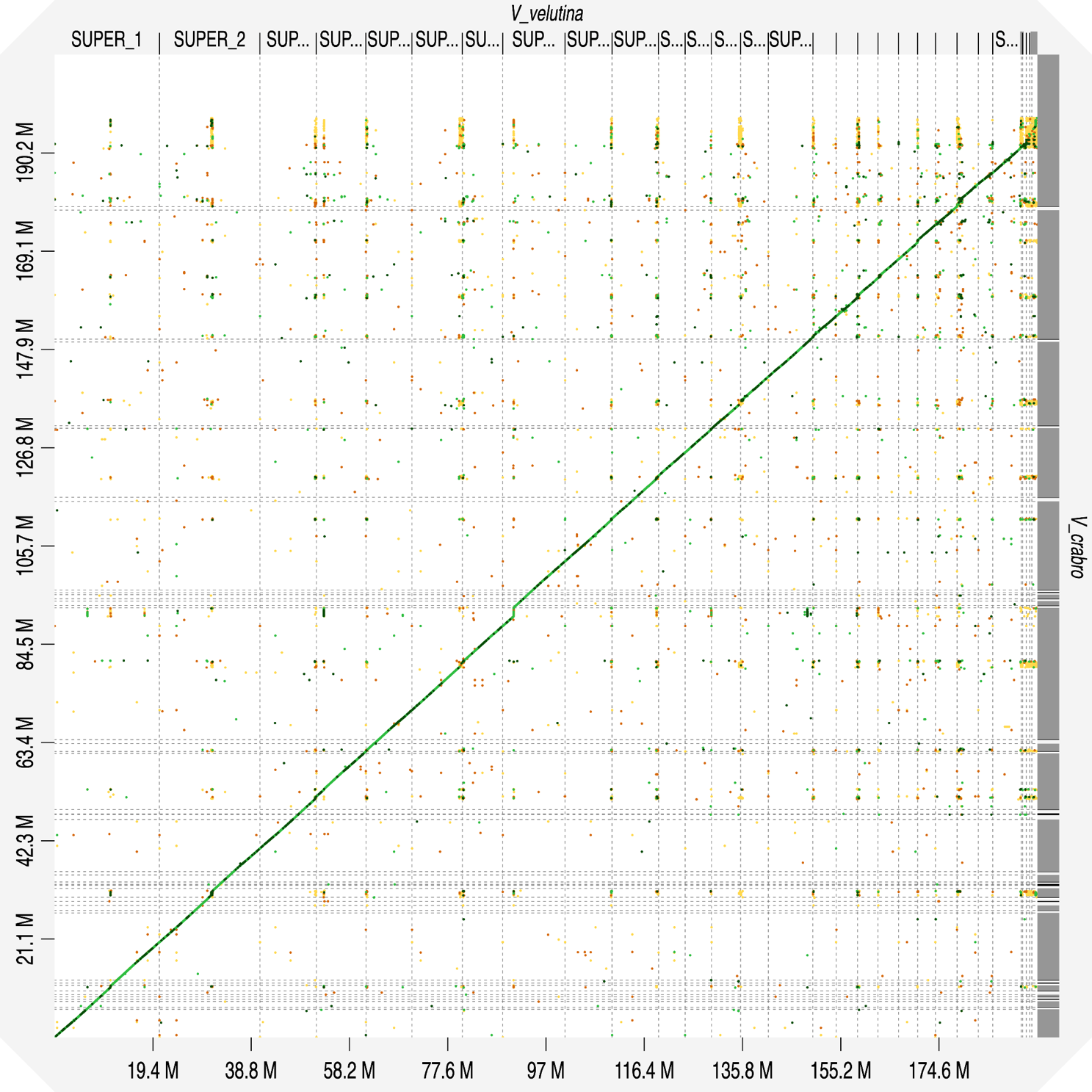


#### Figure SF5: Synteny dot plot comparing *Vespa velutina* and *Vespa crabro*.

DGenies dot plot from minimap2 alignment. *Vespa velutina* contigs are on the top, *V. crabro* contigs are on the right. Each dot represents how similar the two sequences are, based on BLAST-like identity score. Each match is graded from dark green (0.75 to 1, i.e. matching very well), to light green (0.5 to 0.75, i.e. matching well), to orange (0.25 to 0.5, i.e. matching not well), to yellow (0 to 0.25, i.e. matching not well at all).


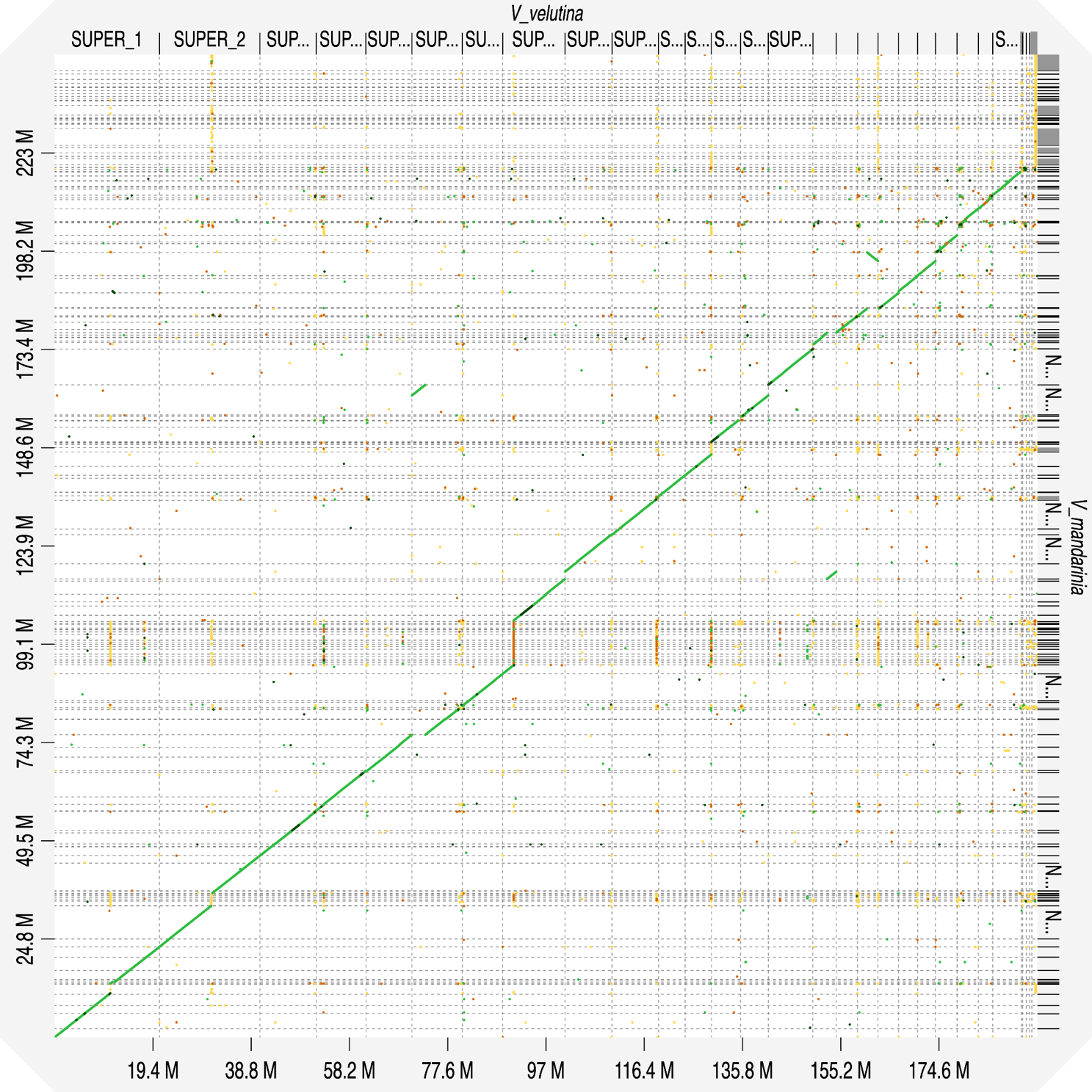


#### Figure SF6: Synteny dot plot comparing *Vespa velutina* and *Vespa mandarinia*.

DGenies dot plot from minimap2 alignment. *Vespa velutina* contigs are on the top, *V. mandarinia* contigs are on the right. Each dot represents how similar the two sequences are, based on BLAST-like identity score. Each match is graded from dark green (0.75 to 1, i.e. matching very well), to light green (0.5 to 0.75, i.e. matching well), to orange (0.25 to 0.5, i.e. matching not well), to yellow (0 to 0.25, i.e. matching not well at all). There is one off-diagonal inversion in *V. mandarinia* (length: 1,152,777 bp, locus NW_023395844.1, start 3701405, end 5336819 on original strand) when mapped to *V. velutina* at locus SUPER_22, start 2372742, end 4003498 on original strand.


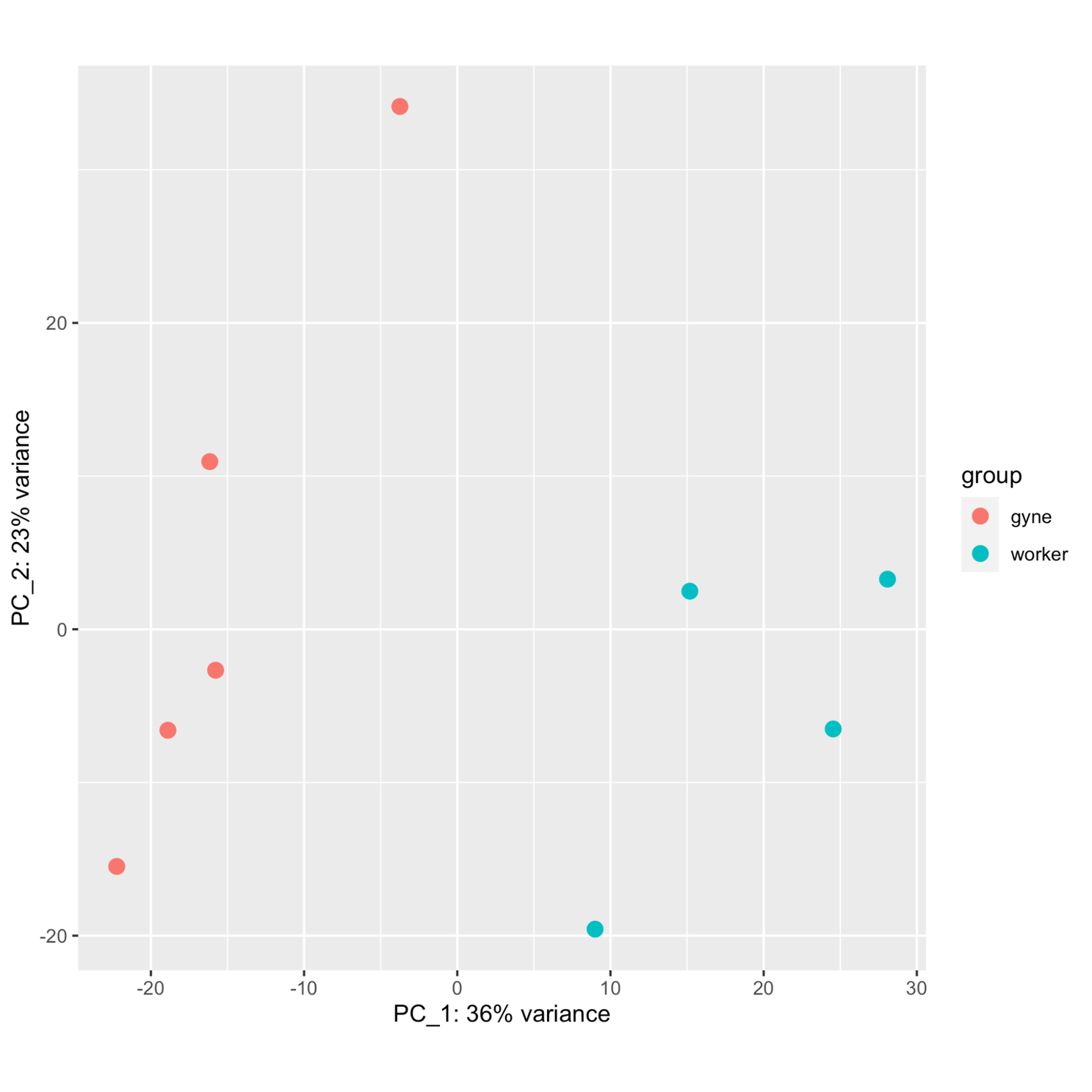


#### Figure SF7: Two First Principal Components of *Vespa crabro* brain gene expression

Gene expression of brains is clearly separated between castes in PC1. PC1 (x axis) explains 36% of the variance between 4 workers (blue dot) and 5 gynes (red dot). PC2 (y axis) explains 23% of the variance between samples.

####
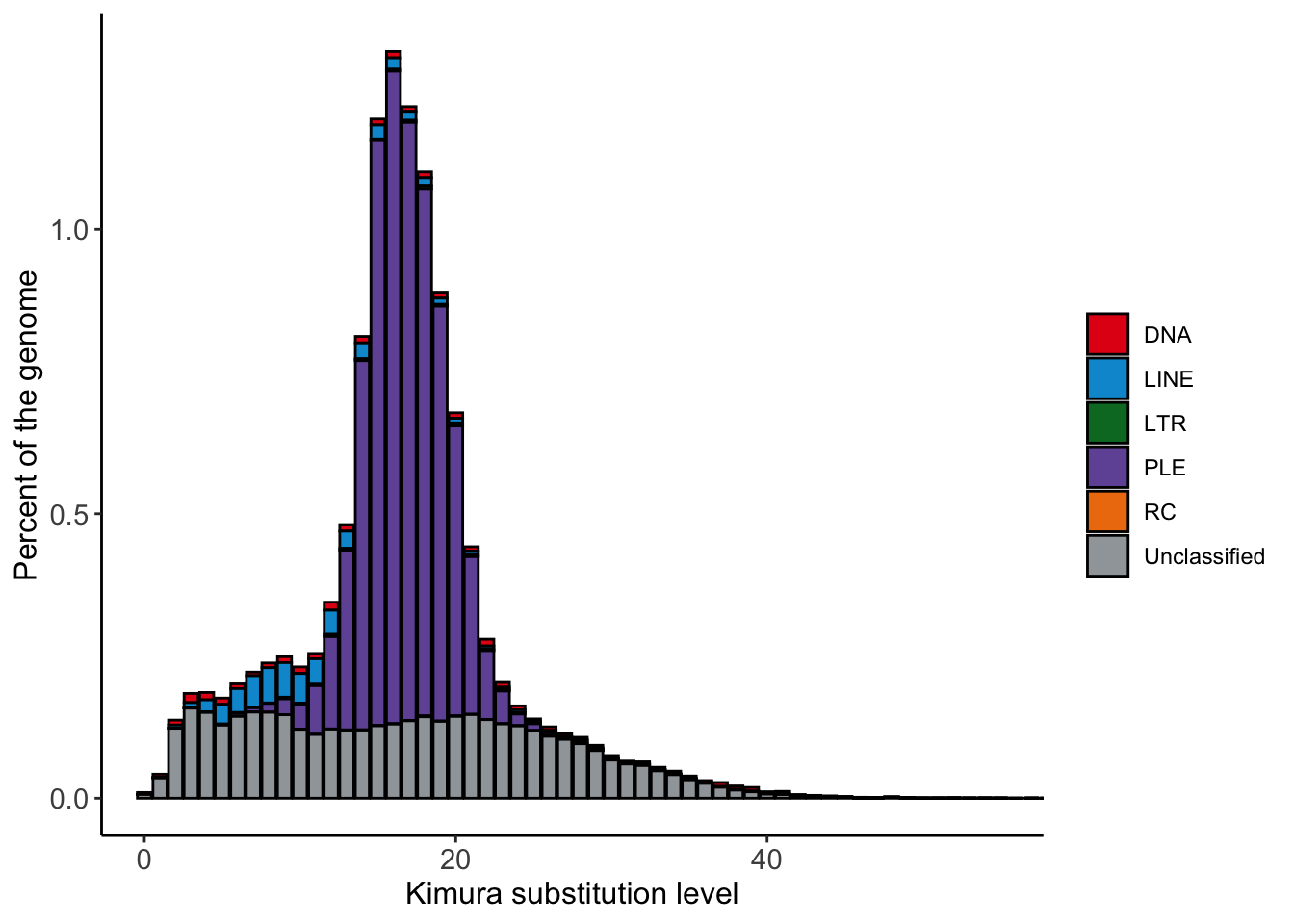
Figure SF8: Transposable Elements Landscape for *Vespa crabro.* Percent of genome coverage for each TE class according to their genetic distance (Kimura 2-parameter). Insertions clustering to the left indicate that copies did not diverge greatly from the consensus and correspond to more recent elements.

This distribution is typical of insect genomes. There is a large proportion of Penelope elements (purple).

####
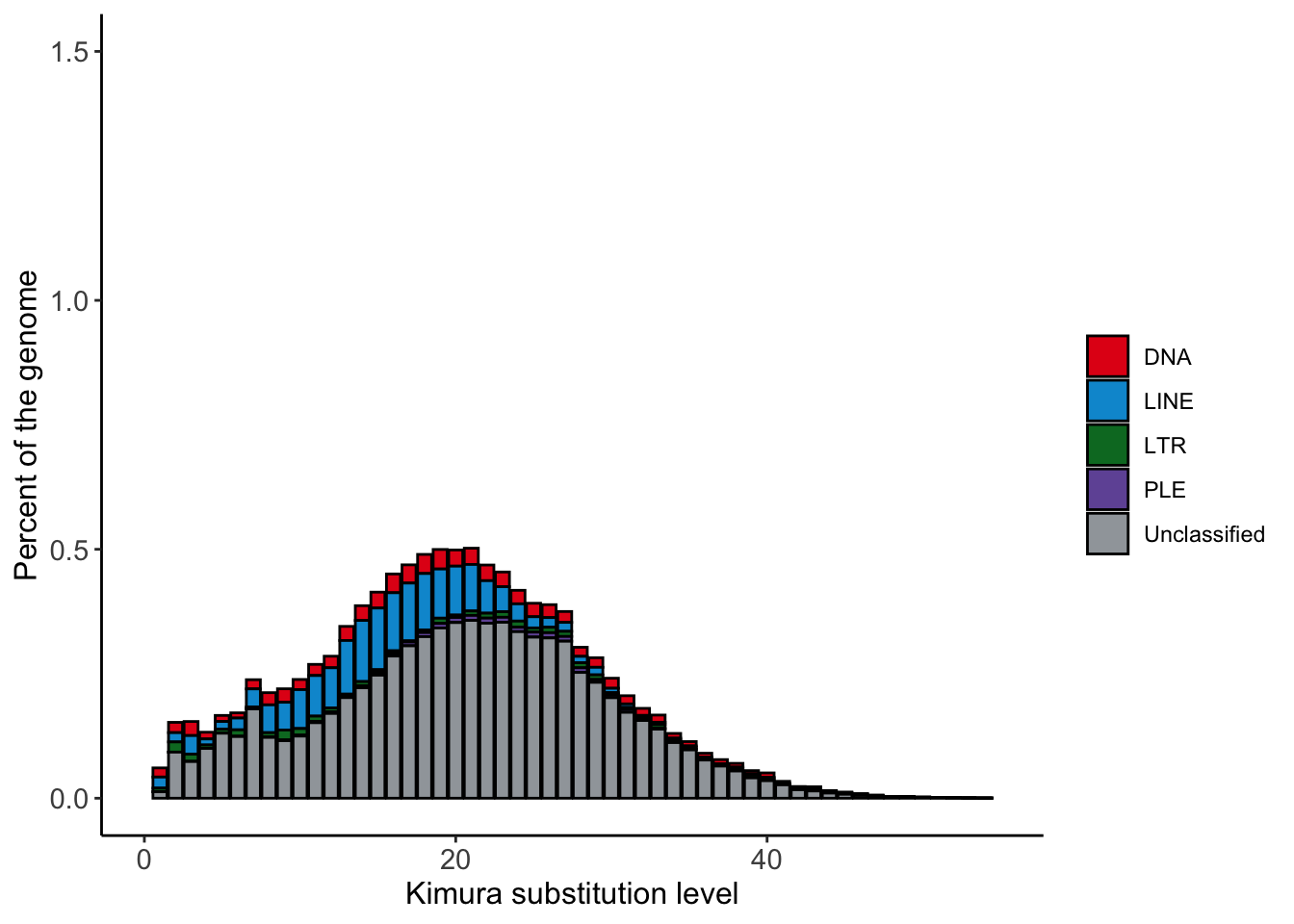
Figure SF9: Transposable Elements Landscape for *Vespa velutin*a. Percent of genome coverage for each TE class according to their genetic distance (Kimura 2-parameter). Insertions clustering to the left indicate that copies did not diverge greatly from the consensus and correspond to more recent elements.

This distribution is typical of insect genomes.

#### Figure SF10: Comparison of Transposable Elements Proportions
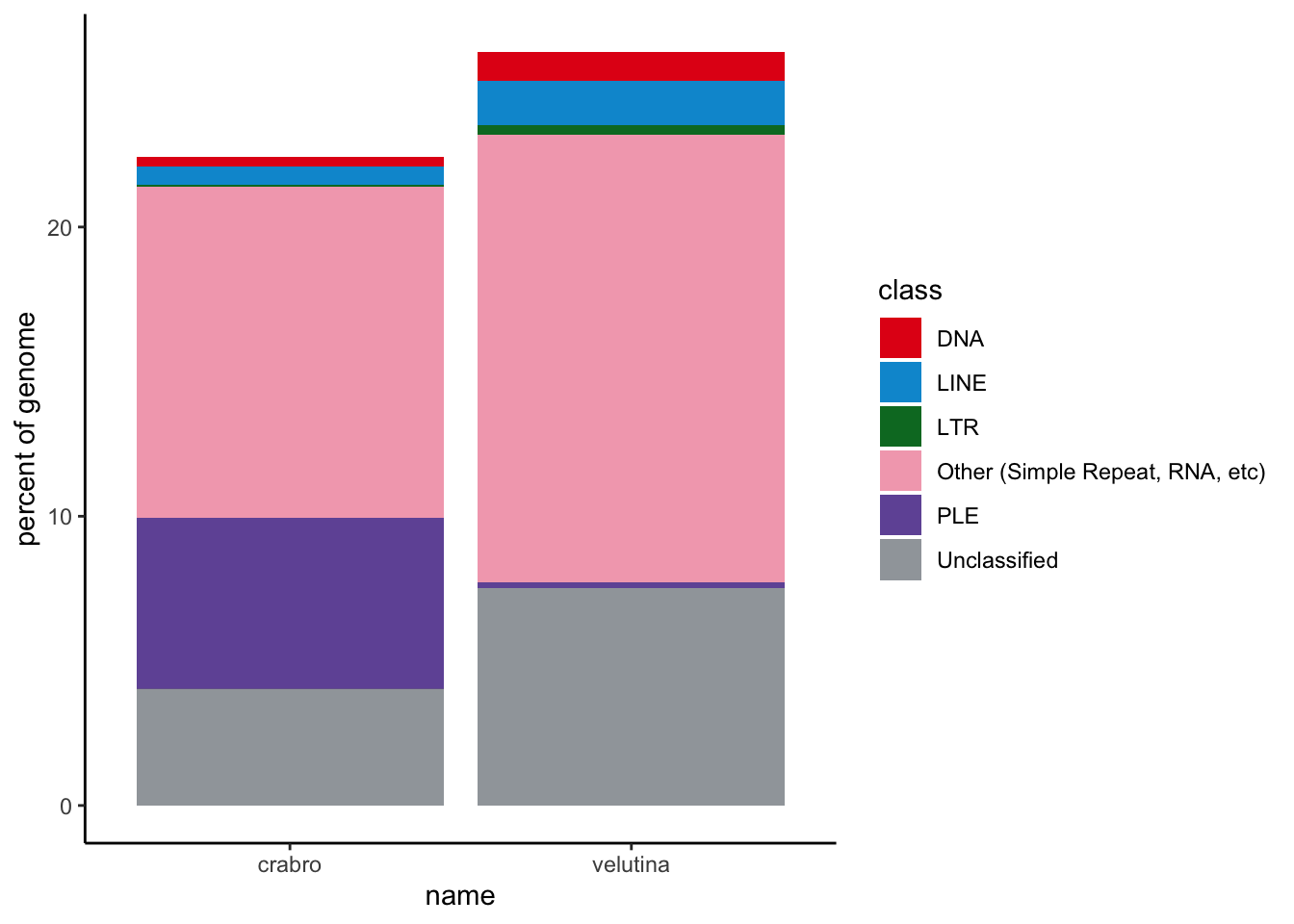


TE in *Vespa velutina* take a larger proportion of the genome (26%) than *Vespa crabro* (22%).

# References

1. Andrews, S. & Others. FastQC: a quality control tool for high throughput sequence data. (2010).

2. Bolger, A. M., Lohse, M. & Usadel, B. Trimmomatic: a flexible trimmer for Illumina sequence data. *Bioinformatics* **30**, 2114–2120 (2014).

3. Luo, R. *et al.* SOAPdenovo2: an empirically improved memory-efficient short-read de novo assembler. *Gigascience* **1**, 18 (2012).

4. Dohm, J. C. *et al.* The genome of the recently domesticated crop plant sugar beet (Beta vulgaris). *Nature* **505**, 546–549 (2014).

5. Langmead, B. & Salzberg, S. L. Fast gapped-read alignment with Bowtie 2. *Nat Methods* **9**, 357–359 (2012).

6. Gurevich, A., Saveliev, V., Vyahhi, N. & Tesler, G. QUAST: quality assessment tool for genome assemblies. *Bioinformatics* **29**, 1072–1075 (2013).

7. Simão, F. A., Waterhouse, R. M., Ioannidis, P., Kriventseva, E. V. & Zdobnov, E. M. BUSCO: assessing genome assembly and annotation completeness with single-copy orthologs. *Bioinformatics* **31**, 3210–3212 (2015).

8. Waterhouse, R. M. *et al.* BUSCO Applications from Quality Assessments to Gene Prediction and Phylogenomics. *Mol Biol Evol* **35**, 543–548 (2018).

9. Marçais, G. & Kingsford, C. A fast, lock-free approach for efficient parallel counting of occurrences of k-mers. *Bioinformatics* **27**, 764–770 (2011).

10. R Core Team. *R: A language and environment for statistical computing*. (2014).

11. Campbell, M. S., Holt, C., Moore, B. & Yandell, M. Genome Annotation and Curation Using MAKER and MAKER-P. *Curr Protoc Bioinforma.* **48**, 4.11.1-39 (2014).

12. Dobin, A. *et al.* STAR: ultrafast universal RNA-seq aligner. *Bioinformatics* **29**, 15–21 (2013).

13. Pertea, M. *et al.* StringTie enables improved reconstruction of a transcriptome from RNA-seq reads. *Nat Biotechnol* **33**, 290–295 (2015).

14. Trapnell, C. *et al.* Differential gene and transcript expression analysis of RNA-seq experiments with TopHat and Cufflinks. *Nat Protoc* **7**, 562–578 (2012).

15. Song, L. & Florea, L. CLASS: constrained transcript assembly of RNA-seq reads. *BMC Bioinformatics* **14 Suppl 5**, S14 (2013).

16. Guttman, M. *et al.* Ab initio reconstruction of cell type–specific transcriptomes in mouse reveals the conserved multi-exonic structure of lincRNAs. *Nat Biotechnol* **28**, 503–510 (2010).

17. Haas, B. J. *et al.* Improving the Arabidopsis genome annotation using maximal transcript alignment assemblies. *Nucleic Acids Res* **31**, 5654–5666 (2003).

18. Haas, B. J. *et al.* De novo transcript sequence reconstruction from RNA-seq using the Trinity platform for reference generation and analysis. *Nat Protoc* **8**, 1494–1512 (2013).

19. Blanco, E., Parra, G. & Guigó, R. Using geneid to identify genes. *Curr Protoc Bioinforma.* **Chapter 4**, Unit 4.3 (2007).

20. Parra, G., Blanco, E. & Guigó, R. GeneID in Drosophila. *Genome Res* **10**, 511–515 (2000).

21. Majoros, W. H., Pertea, M. & Salzberg, S. L. TigrScan and GlimmerHMM: two open source ab initio eukaryotic gene-finders. *Bioinformatics* **20**, 2878–2879 (2004).

22. Lomsadze, A., Ter-Hovhannisyan, V., Chernoff, Y. O. & Borodovsky, M. Gene identification in novel eukaryotic genomes by self-training algorithm. *Nucleic Acids Res* **33**, 6494–6506 (2005).

23. Stanke, M. *et al.* AUGUSTUS: ab initio prediction of alternative transcripts. *Nucleic Acids Res* **34**, W435-9 (2006).

24. Korf, I. Gene finding in novel genomes. *BMC Bioinformatics* **5**, 59 (2004).

25. Haas, B. J. *et al.* Automated eukaryotic gene structure annotation using EVidenceModeler and the Program to Assemble Spliced Alignments. *Genome Biol* **9**, R7 (2008).

26. Iwata, H. & Gotoh, O. Benchmarking spliced alignment programs including Spaln2, an extended version of Spaln that incorporates additional species-specific features. *Nucleic Acids Res* **40**, e161 (2012).

27. Slater, G. S. C. & Birney, E. Automated generation of heuristics for biological sequence comparison. *BMC Bioinformatics* **6**, 31 (2005).

28. Chin, C.-S. *et al.* Phased diploid genome assembly with single-molecule real-time sequencing. *Nat Methods* **13**, 1050–1054 (2016).

29. Ghurye, J., Pop, M., Koren, S., Bickhart, D. & Chin, C.-S. Scaffolding of long read assemblies using long range contact information. *BMC Genomics* **18**, 527 (2017).

30. Garrison, E. & G., M. Haplotype-based variant detection from short-read sequencing. *ArXiv Prepr.* (2012) doi:arXiv:1207.3907 [q-bio.GN].

31. Chow, W. *et al.* gEVAL — a web-based browser for evaluating genome assemblies. *Bioinformatics* **32**, 2508–2510 (2016).

32. Blum, M. *et al.* The InterPro protein families and domains database: 20 years on. *Nucleic Acids Res* **49**, D344–D354 (2021).

33. Törönen, P., Medlar, A. & Holm, L. PANNZER2: a rapid functional annotation web server. *Nucleic Acids Res* **46**, W84–W88 (2018).

34. Götz, S. *et al.* High-throughput functional annotation and data mining with the Blast2GO suite. *Nucleic Acids Res* **36**, 3420–3435 (2008).

35. Almagro Armenteros, J. J. *et al.* SignalP 5.0 improves signal peptide predictions using deep neural networks. *Nat Biotechnol* **37**, 420–423 (2019).

36. Lu, S. *et al.* CDD/SPARCLE: the conserved domain database in 2020. *Nucleic Acids Res* **48**, D265–D268 (2020).

37. Jones, P. *et al.* InterProScan 5: genome-scale protein function classification. *Bioinformatics* **30**, 1236–1240 (2014).

38. Huntley, R. P. *et al.* The GOA database: gene Ontology annotation updates for 2015. *Nucleic Acids Res* **43**, D1057-63 (2015).

39. Moriya, Y., Itoh, M., Okuda, S., Yoshizawa, A. C. & Kanehisa, M. KAAS: an automatic genome annotation and pathway reconstruction server. *Nucleic Acids Res* **35**, W182-5 (2007).

40. Kanehisa, M. & Goto, S. KEGG: kyoto encyclopedia of genes and genomes. *Nucleic Acids Res* **28**, 27–30 (2000).

41. Hoshiba, H., Matsuura, M. & Imai, H. T. Karyotype evolution in the social wasps Hymenoptera, Vespidae. *Jpn. J. Genet.* **64**, 209–222 (1989).

42. Hoshiba, H. & Yamamoto, H. Karyological Studies on the Three Species of the Haploid Males of Vespinae, Vespa mandarinia Smith, V. simillima xanthoptera Cameron and Vespula flaviceps (Smith) (Vespidae, Hymenoptera). *Proc Jpn Acad Ser B Phys Biol Sci* **61**, 67–70 (1985).
